# Modular Mass Spectrometric Tool for Analysis of Composition and Phosphorylation of Protein Complexes

**DOI:** 10.1371/journal.pone.0000358

**Published:** 2007-04-04

**Authors:** Justin D. Blethrow, Chao Tang, Changhui Deng, Andrew N. Krutchinsky

**Affiliations:** 1 Department of Cellular and Molecular Pharmacology, University of California San Francisco, San Francisco, California, United States of America; 2 XProteo Inc, New York, New York, United States of America; 3 Department of Pharmaceutical Chemistry, University of California San Francisco, San Francisco, California, United States of America; Wellcome Trust Sanger Institute, United Kingdom

## Abstract

The combination of high accuracy, sensitivity and speed of single and multiple-stage mass spectrometric analyses enables the collection of comprehensive sets of data containing detailed information about complex biological samples. To achieve these properties, we combined two high-performance matrix-assisted laser desorption ionization mass analyzers in one modular mass spectrometric tool, and applied this tool for dissecting the composition and post-translational modifications of protein complexes. As an example of this approach, we here present studies of the *Saccharomyces cerevisiae* anaphase-promoting complexes (APC) and elucidation of phosphorylation sites on its components. In general, the modular concept we describe could be useful for assembling mass spectrometers operating with both matrix-assisted laser desorption ionization (MALDI) and electrospray ionization (ESI) ion sources into powerful mass spectrometric tools for the comprehensive analysis of complex biological samples.

## Introduction

**Figure 1:**
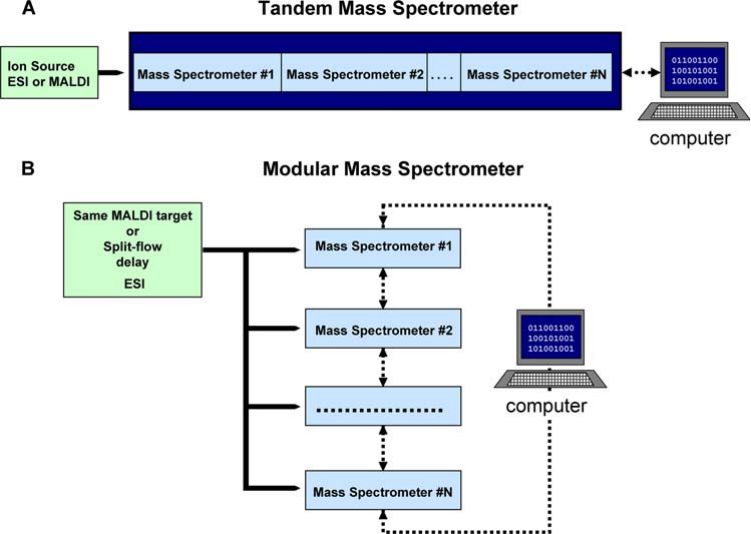
Schematic diagrams of (A) a tandem mass spectrometer, and (B) a modular mass spectrometer.

In recent years, mass spectrometric (MS) analysis of biological samples has increasingly entailed direct analysis of complex protein mixtures, often with the objective of detailed characterization of the various components. This trend toward ever greater sample complexity has been enabled and in turn driven by the rapid development of powerful mass spectrometric tools. A general characteristic of recent mass spectrometers is that most are composed of a sequence of multiple mass analyzers with different strengths and properties, resulting in tandem instruments that possess capabilities unattainable by the individual components (**Fig. 1A**). For example, triple quadrupole (QQQ) [1], [2], Quadrupole/Time-of-flight (QTOF or QqTOF) [3], Quadrupole/Ion trap (QTRAP) [4], Time-of-flight/Time-of-flight (TOF-TOF) [5] hybrid tandem mass spectrometers have all proven to be powerful tools for analytical research.

Tandem instruments can combine high mass accuracy with high-speed measurement, greatly facilitating the analysis of complex mixtures. For example, the addition of Time-of-flight (TOF), Fourier Transform Ion Cyclotron Resonance (FT-ICR) and Orbitrap mass analyzers to an ion trap (IT) has greatly increased the accuracy of measurements during the multiple stages of mass spectrometric (MS^n^) analysis [6]–[9]. Physical assembly of the two types of mass spectrometers couples their performances, providing a fast link between precursor ion selection steps and subsequent MS^n^ experiments on the selected ions [10], [11]. This option is advantageous when speed and accuracy are crucial for the success of analysis, as it is, for example, when the mass spectrometer is coupled on-line to an HPLC system [12], [13].

Physical coupling of multiple mass spectrometers in tandem has some disadvantages. Optimal operation conditions for different mass spectrometers and modes of operation of a tandem instrument may differ significantly, producing the need to compromise in the performance of one mass spectrometer at the expense of another [14]–[16]. Decoupling the parts of a hybrid instrument is one solution to this problem. Indeed, a modular mass spectrometric tool can be assembled from several mass spectrometers without physically coupling them in one instrument. Several mass spectrometers can be used as separate modules, fine-tuned for each particular type of analysis, and applied in turn to extract comprehensive information about the sample in a data-dependent manner. The collected data can be analyzed quickly by a computer, which generates a set of instructions based on the results of analysis of the data obtained in the previous instrument and passes them to the next one. Theoretical speed of the analysis in such a modular tool is only limited by the speed of the sample analysis in the different instruments and the speed of transfer of the remaining part of the sample from one mass spectrometer to another.

A schematic diagram of a modular instrument based on this concept is illustrated in **Figure 1B**. Multiple MALDI instruments can easily be combined into one system through the use of interchangeable MALDI target plates. Similarly, multiple ESI-based mass spectrometers can be coupled by using a split-flow technique to introduce a delay between sample arrival times at the ion sources of different instruments. In this case, the time-delay should be greater than the duty cycle of the upstream instrument so that data-dependent instructions can be generated and transmitted to the downstream instrument.

This concept has been used to combine a high resolution, high mass accuracy MALDI-QqTOF [17] instrument with a high-speed, high-sensitivity MALDI-IT [18] mass spectrometer. This combination has proven to be extremely useful for gaining insight into many challenging biological problems [19]–[22]. Initial studies of the utility of this instrument combination utilized in-house modified instruments. However, the recent commercial introduction of similar mass spectrometers has opened the possibility to reproduce this approach in any laboratory.

This paper describes a modular mass spectrometric tool based on two MALDI mass spectrometers, the proTOF [23] (PerkinElmer) and the vMALDI-LTQ[24] (Thermo Electron). We demonstrate the utility of this tool for studying the composition of protein complexes and for identifying the phosphorylation sites on the subunits of the *S. cerevisiae* anaphase promoting complex (APC) [25].

## Results

### Combined performance of the mass spectrometers

As a first step in the development of our combined mass spectrometer system, we designed a magnetic MALDI target that can be exchanged between prOTOF and vMALDI-IT instruments. The target permits sequential analysis of unique samples using these two instruments (see Methods section).

**Figure 2:**
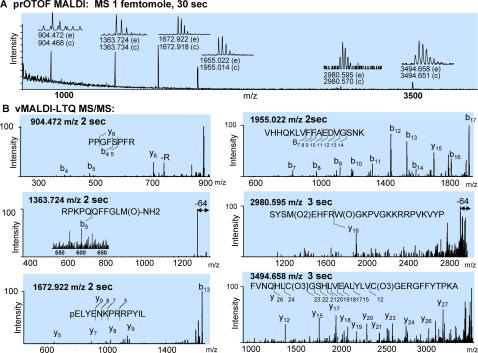
Combined performances of a prOTOF and a vMALDI-LTQ mass spectrometers as one modular tool. (A) prOTOF-MALDI-MS spectrum of a 1 femtomole mixture of six peptides, obtained in 30 seconds of spectrum acquisition time. The measurements of the m/z values of the peptides were performed using an external instrument calibration. The monoisotopic resolution for the detected ion peaks as well as the calculated (c) and the experimental (e) m/z values are shown. The peptides are bradykinin (fragment 2–9: PPGFSPFR, m/z = 904.468, theoretical value), Substance P (RPKPQQFFGLM-NH2, m/z = 1347.736), neurotensin (pELYENKPRRPYIL, m/z = 1672.918), amyloid β-protein (fragment 12–28: VHHQKLVFFAEDVGSNK, m/z = 1955.014), ACTH (SYSMEHFRWGKPVGKKRRPVKVYP, m/z = 2932.588), and B chain of oxidized insulin (FVNQHLC(O3)GSHLVEALYLVC(O3)GERGFFYTPKA, m/z = 3494.651). (B) vMALDI-LTQ MS/MS spectra of all 6 detected peptides. All spectra were measured in 2–3 seconds after the automatic collection of the MS/MS spectra. The interpretation of the observed fragmentation spectra and the identity of the observed peptides are indicated in each panel.

We analyzed a mixture of six known peptides at the single femtomole scale to evaluate the performance of our combined mass spectrometer system. The first step in this analysis was the rapid collection of a high resolution, high mass accuracy MS spectrum using the prOTOF instrument. A ProTOF mass spectrometer is a one-task instrument for rapid measurement of single-stage MS spectra [23]. **Figure 2A** shows an MS spectrum of our peptide mixture obtained in a thirty second acquisition. At least six peaks were observed with signal-to-noise ratios above 1.5∶1. The observed resolution is greater than 10,000, enabling clear determination of peptide isotopic distributions. Importantly, the mass accuracy was within a few parts per million, even for statistically weak signals. Only a minuscule amount of sample was consumed during this first step in the analysis.

The first-stage MS spectrum was used to generate a list of targets for direct MS^n^ analysis using the second instrument, vMALDI-LTQ. An LTQ mass spectrometer is an extremely fast and efficient device for acquisition of MS^n^ data [26], [27]. Peaks meeting user-defined criteria are automatically extracted to a text file; this file is used to generate an instrument control script to automate acquisition of the fragmentation spectra in the second instrument. The LTQ instrument iterates through this peak list; in each cycle, the desired precursor ions are selected for subsequent fragmentation and product ion analysis. **Figure 2B** shows MS/MS spectra acquired for the six peptides detected in the 1 fmol sample. Each tandem spectrum was acquired over two to three seconds, for a total collection time of about fifteen seconds. The product spectra exhibit a variable degree of fragmentation, with fragmentation pathways typical of singly charged peptides [28], [18]. The m/z values of two peptides and their fragmentation spectra suggest that they contained oxidized methionine and tryptophan residues (MS/MS of 1363.724 m/z and 2980.595 m/z). Both spectra show prominent fragments representing neutral loss of 64 Da from the precursor ions, arising from loss of CH_3_SOH from methionine sulfoxide. Higher order MS^n^ spectra of those fragments which lost a neutral group frequently contain information about the identity and exact position of the modification sites [29], [30].

The successful analysis of complex peptide mixtures is made far more likely if one is able to acquire both high accuracy precursor ion masses and comprehensive fragmentation data [10], [11], [31]. We achieve these properties through the combined use of two physically decoupled instruments, each optimized for a specific role. In combination, these spectrometers provide data containing 5–10 parts-per-million (ppm) accuracy in precursor mass measurement and informative fragmentation spectra while readily functioning in the single femtomole range and requiring only 2–3 seconds per MS/MS spectrum.

### Analysis of proteins in the *S. cerevisiae* APC complex

**Figure 3:**
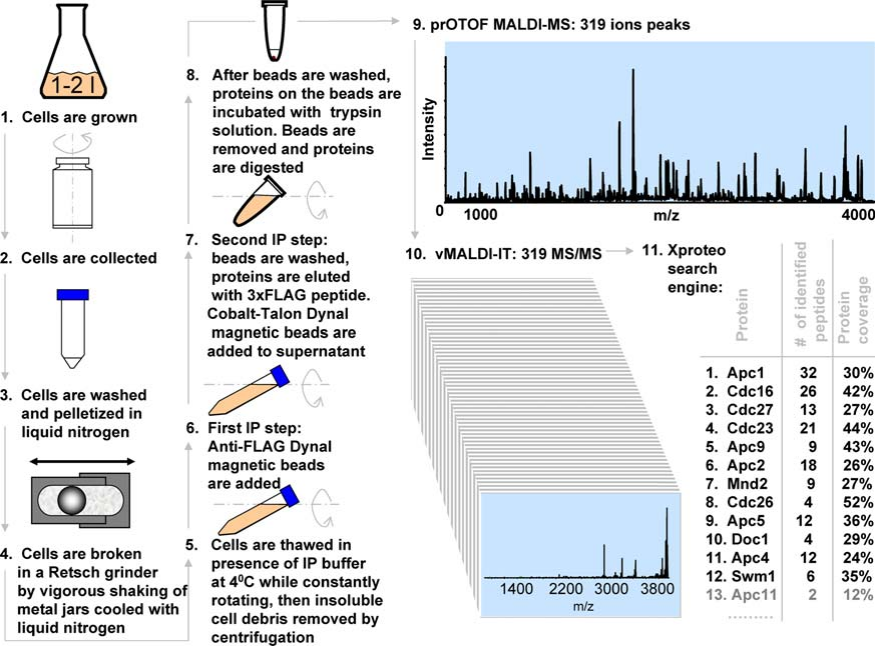
The flow of steps in the experiments for characterization of the subunits of the S. cerevisiae APC with a modular mass spectrometric tool.

To demonstrate the usefulness of our method to the characterization of complex biological samples, we analyzed the protein composition and post-translational modifications of the *Saccaromyces cerevisiae* anaphase-promoting complex [25]. **Figure 3** shows the flow of steps in our experiments for characterization of APC immunopurified from *S. cerevisiae*. The first few steps are typical for the immunopurification of stably-associated protein complexes from unsynchronized cell cultures using proteins genomically tagged with an epitope at the C-terminus [32], [33]. The characteristic feature of our approach is that we use a 3xFLAG-hexahistidine (3xFLAG-6xH) tandem affinity tag [34]. The choice of the 3xFLAG-6xH epitope is based on several practical considerations. A high quality monoclonal anti-FLAG antibody is commercially available (M2, Sigma-Aldrich). This reagent enables us to achieve high specificity and yield in the initial purification step, and provides the ability to quickly and efficiently elute the complexes under non-denaturing conditions by competition with a 3xFLAG peptide. The single imunopurification step utilizing a 3xFLAG tag is usually sufficient to obtain highly enriched protein complexes. However, an additional tandem purification step can be very useful to remove an excess of the elution peptide and concentrate the complexes on the metal chelating resin. We perform the second step very quickly, within 10–15 minutes, to minimize possible losses of proteins from the complexes [22]. After the final wash of the chelating resin, the purified proteins can be efficiently eluted or left on the beads as needed.

Proteins co-purifying with the APC subunit Cdc16-3xFLAG-6xH were enriched according to the scheme in Figure 3, starting with one liter of yeast culture grown to a density of (2–4)×10^7^ cells/ml. After the final wash (Step 8 of **Fig. 3**), a trypsin solution was added directly to the Co-Talon magnetic beads, and the slurry was gently mixed and incubated at 37 C for 5 min. After 5 minutes, the beads were separated from the supernatant with a magnet; the solution was then collected and incubated at 37 C for 5 hours to digest all proteins that came off the beads. In a separate line of experiments, we found that a brief (∼5 min) incubation of the beads with trypsin elutes most of the proteins associated in noncovalent complexes, while possibly minimizing liberation of contaminant proteins associated tightly but, presumably, nonspecifically with the beads (see supplementary **Figure S1**). We currently investigate whether limited proteolysis can be used for the selective elution of the protein complexes from the beads.

The tryptic peptides were directly analyzed in the prOTOF mass spectrometer without further purification or fractionation steps. At least 319 ion peaks with a signal-to-noise ratio above 1.2:1 were detected in the MS spectrum. These peaks were selected for MS/MS analysis, and their m/z values were used to generate a vMALDI-IT acquisition script. The average acquisition time was approximately three seconds per MS/MS spectrum, resulting in a total measurement time of twenty minutes. The combined data were converted to DTA format [35] and supplied to the XProteo search engine (www.Xproteo.com). Searching of the *S. cerevisiae* data base (NCBI non-redundant data base version 07/06/06) resulted in identification of all 13 proteins known to stably comprise the APC complex [25] (**Fig. 3**). Twelve identifications surpassed the probability threshold for 99% confidence at a false alarm rate of 0.5% (For complete information about the XProteo search scores please refer to the “FAQ” section at the www.xproteo.com). Out of 319 detected ion peaks, 168 were identified with the APC components. For Apc11, the 13^th^ component of the APC, only two peptides were identified, resulting in 12% coverage of this small (19 kDa) protein. Examination of the two MS/MS spectra assigned as peptides from Apc11 indicated an accurate match between the experimental and theoretical m/z values of parent ions to within a few parts-per-million. In addition, both peptides displayed dominant fragmentation events consistent with the predicted positions of aspartic and glutamic acid residues [28], [18]. Thus, both MS and MS/MS spectra confirmed the identification of the Apc11 protein. The XProteo report containing the parameters of the search and the complete results can be found in the supplementary **Report S1** online.

A control experiment was performed in parallel using an equal amount of yeast cells not expressing any tagged protein. This resulted in the identification of several contaminant proteins (see supplementary **Figure S2** and supplementary **Report S2** online). Although our tandem purification procedure greatly reduces the presence of background proteins, we can still identify, on average, 2–6 contaminating proteins in the control samples. Most of these proteins are highly abundant proteins in the cell (e.g. Fks1, Tef1, Pho84, Adh1, Tdh1, Uba4), which presumably bind either the beads or the protein complexes nonspecifically. Uba4 contains a sequence (IYKDDE; amino acids 333–338) that resembles a FLAG epitope (DYKXXD) [36]. When the purified APC proteins are separated by SDS-PAGE and the entire gel lane is processed by in-gel digestion procedure (see supplementary **Figure S3**), we frequently identify a background of a small number of the same proteins. We plan to use new emerging techniques to distinguish and reduce the interference from non-specifically interacting proteins [37].

We compared our MS identification methodology with a more conventional protocol employing a QTRAP mass spectrometer (Sciex) coupled by online electrospray ionization to a nano-HPLC system running at 150 nl/min. 274 MS/MS spectra obtained over the course of 2-hour gradient separation were analyzed with the MASCOT search engine, resulting in identification of all 13 APC subunits (see supplementary **Report S3**). We also identified several peptides originating from probable sample impurities, including two peptides derived from Glyceraldehyde-3-phosphate dehydrogenase (Tdh1), one of the most abundant proteins in the cell [38].

### Analysis of APC phosphorylation

Phosphopeptides display a characteristic fragmentation pattern in MS/MS analysis, commonly exhibiting a predominant ∼98 Da reduction in mass due to neutral loss of phosphoric acid [39], [28]. We examined our APC MS/MS data set for the presence of peptides displaying this pattern. As a first pass analysis, we plotted the MS/MS ion intensity at m/z-98 for all obtained precursor m/z values, using the “Ion Map” function of the Xcalibur Qual Browser program (Thermo-Finnigan). However, this method tends to produce false positives when particularly abundant precursors happen to yield a minor fragment at m/z-98. Similarly, low abundance precursor phosphopeptides may be missed due to low relative MS/MS signal intensities. We found that plotting the signal-to-noise ratio of the peak at m/z-98 rather than the raw signal intensity is a more reliable way of detecting a significant neutral loss. To this end, we wrote our own small program that plots the signal-to-noise ratios for all candidate neutral loss peaks in an MS/MS data set as a function of the m/z of their precursor ions.

**Figure 4:**
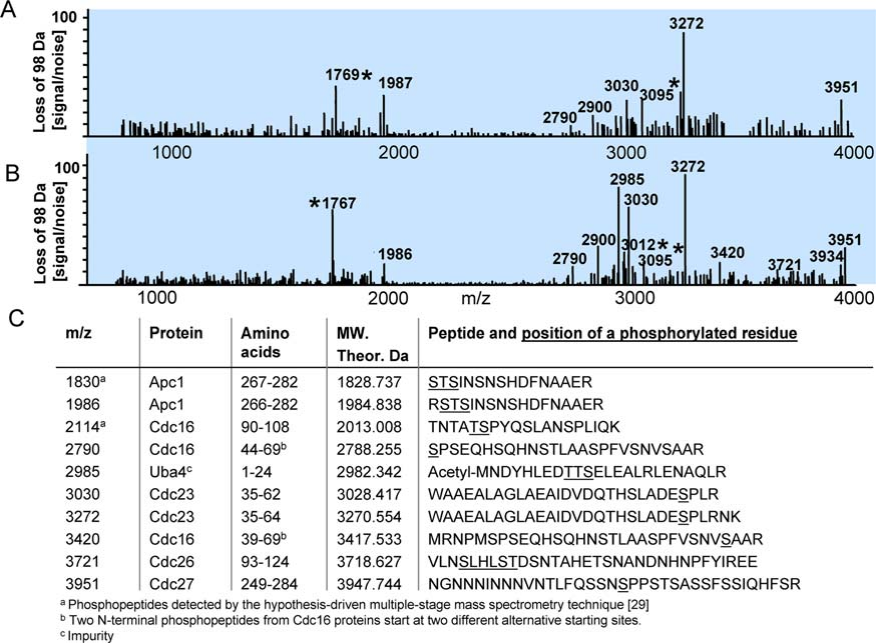
(A) A map of neutral losses of 98 Da detected in all MS/MS spectra obtained from the sample shown in Fig. 3. The APC was purified from ∼(2–4)×10^10^ cells. (B) A map of neutral losses of 98 Da detected in all MS/MS spectra obtained from APC purified from a large culture of about 10^11^ cells. All peaks with signal-to-noise ratios above 10-to-1 were examined by MS/MS and MS/MS/MS to confirm the loss of 98 Da and to identify the phosphopeptides and plausible position of a phosphate group. Peaks indicated with an asterisk were not identified. (C) Summary of the identified phosphopeptides from proteins co-immunopurified with the Cdc16-3xFlag-6xH protein.

**Figure 4A** shows a map of neutral losses of 98 Da for all MS/MS spectra from the APC preparation shown in Figure 3. To improve the detection of low-stoichiometry phosphorylation sites we repeated the APC immunopurification several times, and increased the amount of yeast cells from (2–4)×10^10^ to ∼10^11^. **Figure 4B** shows the map of neutral losses of 98 Da obtained from one of these larger samples. In this case, we performed MS/MS on 419 detected precursors and analyzed the fragmentation spectra to produce the map of losses of 98 Da. There is good reproducibility of the relative intensities of the detected phosphopeptides in both maps. To map the locations of phosphorylation sites, we acquired MS^3^ spectra of all species exhibiting a loss of 98 Da with a signal-to-noise ratio above ∼10. The results of this analysis are summarized in **Figure 4C**.

**Figure 5:**
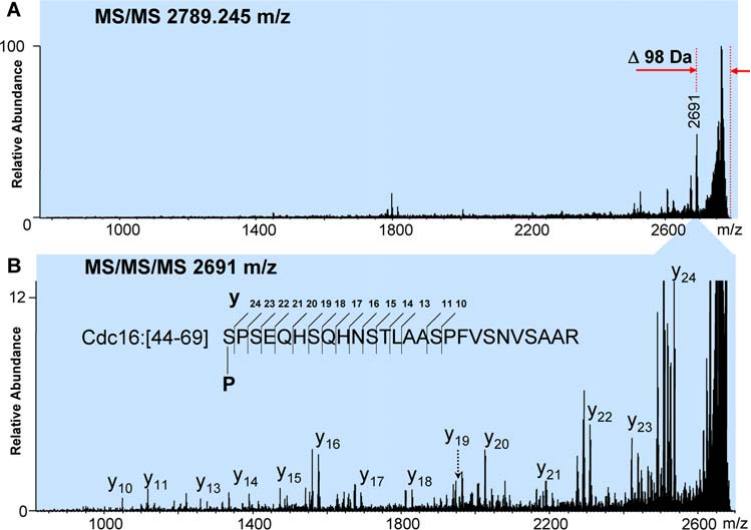
(A) An MS/MS spectrum of a phosphopeptide detected at m/z 2789.245. The loss of 98 Da, which is a specific signature of phosphorylation, is indicated. (B) MS/MS/MS of the fragment that lost 98 Da. The identity of the phosphopeptide and the plausible location of the residue which lost the phosphate group are shown in the panel.

**Figure 5** shows an example of the analysis of a phosphopeptide detected at m/z 2789.245. The MS/MS spectrum of the peptide exhibits a clear loss of 98 Da. MS^3^ analysis of this neutral loss species gave facile fragmentation along the peptide backbone. We also recorded an MS/MS spectrum of the non-phosphorylated form of the peptide at m/z 2709.360, which revealed a fragmentation pattern similar to the fragmentation pattern of the phosphopeptide (**Fig 5B**). Despite good quality of high signal-to-noise fragmentation spectra, the computer search did not produce any candidate protein from which these peptides originated. The search was performed with an assumption that all peptides are tryptic. However, manual interpretation of the spectra and the search among non-tryptic peptides resulted in identification of a “semi-tryptic” peptide (SPSEQHSQHNSTLAASPFVSNVSAAR, residues 44–69) positioned between Met 43 and Thr 70 in the Cdc16 protein. Because this peptide was apparently cleaved with trypsin only once, at the C-terminus, we speculate that the identified peptide represents the N-terminal portion of an alternative form of Cdc16 derived from an internal start codon, with subsequent removal of the methionine. Further interpretation of the MS^3^ spectrum indicates that, most probably, the first amino acid of the peptide, Ser44, is phosphorylated. We also found two Cdc16 peptides derived from a second putative alternate N-terminus (MRNPMSPSEQHSQHNSTLAASPFVSNVSAAR, residues 39–69, and its shorter version NPMSPSEQHSQHNSTLAASPFVSNVSAAR, residues 41–69). Both peptides are also phosphorylated, but seemingly on different residues, according to our analysis of the MS^3^ spectra. In this form of the protein the presumptive initiation Met39 was not processed. We have not yet observed any tryptic peptides from the canonical N-terminal portion of the protein, residues 1–36. The MS^n^ spectra and their interpretation can be found in the supplementary **Figure S4** online.

## Discussion

We combined two high-performance MALDI mass spectrometers, each with its own analytical strengths, into one mass spectrometric tool capable of providing fast, accurate, and sensitive analysis of complex biological samples (**Fig. 1B**). The two instruments are used to sequentially interrogate samples following their deposition on an interchangeable MALDI target. The analysis starts with acquisition of a single-stage MS spectrum at high mass accuracy (5–10 ppm) using the orthogonal Time-of-Flight instrument. Mass values extracted from this survey spectrum are used to generate an instrument control script for subsequent acquisition of MS^n^ data using the LTQ ion trap, an instrument optimized for very high sensitivity fragmentation analysis. This instrument is capable of fast (∼3 sec per spectrum) acquisition of MS/MS spectra in the single femtomole regime, allowing generation of 1200 spectra per hour. At the end of a typical pair of MS and MS/MS analysis runs, the interrogated sample is only partially depleted. Thus, the MS/MS data can be examined for the presence of characteristic fragmentation patterns, such as the neutral loss of 98 from a phosphorylated serine or threonine, and then the sample may be further analyzed by MS^3^ to determine structural information about the selected analytes. In this third pass, we can use longer spectrum acquisition times, up to several minutes, to collect statistically well defined MS^3^ spectra from low-abundance targets. Interpretation of the MS/MS and MS^n^ fragmentation spectra frequently allows identification of the peptides and plausible localization of the phosphate group.

We applied our strategy to the study of the *S. cerevisiae* APC. To check the performance of the modular mass spectrometric tool, we analyzed the unfractionated tryptic peptide mixture obtained after digesting immunopurified proteins directly on the beads. All 13 core components of the APC complex were quickly, robustly and reproducibly identified with this tool. A part of the same sample was also analyzed in an HPLC-QTRAP mass spectrometer, which confirmed the result.

Analysis of cell-cycle averaged phosphorylation of APC subunits resulted in mapping of several phosphorylation sites (**Fig. 4C**). We found that Cdc16, Cdc23 and Cdc27 are phosphorylated on several predicted Cdk1 sites [40]. In addition, we found phosphorylation sites on Apc1 and Cdc26. In the latter case, we were able to determine the position of the phosphorylation sites with accuracy to within several amino acid residues.

The regions of yeast APC subunits harboring found phosphorylation sites are not conserved in their human counterparts [41]. For example, the N-terminal portion of Cdc16 is conserved only within a small family of yeasts. Moreover, we have found that Cdc16 has at least two different N-termini. We did not find peptides from the canonical initiator methionine of Cdc16; instead, we found that one sub-population of the protein starts at Met39 and the other starts at Met43, which is later processed. We also found that Ser44 is sometimes phosphorylated, resulting in a sub-population of Cdc16 that is phosphorylated at the first amino acid residue. It is an interesting question whether phosphorylation of the Ser44 (and another N-terminal peptide, TNTATSPYQSLANSPLIQK, residues 90–108) affects the processing of the N-terminal portion of the protein. We plan to investigate whether there is a functional significance of this phenomenon.

Presently, we looked only at the cell cycle average phosphorylation of the yeast APC, and thus could have missed other phosphorylation sites whose abundance reaches maximum during mitosis. A previous study of human APC phosphorylation revealed a substantial difference in the number of detected phosphorylation sites, six on the two subunits, Apc1 and Apc5, during the S-phase, and 50 sites on the nine subunits, Apc1, Apc2, Cdc27, Apc4, Apc5, Cdc16, Apc7, Cdc23 and Cdc20, during mitosis [41]. We plan to extend our current studies to the analysis of dynamic changes in APC composition and modification over the course of the cell cycle. The ability to rapidly prepare and analyze highly purified native complexes from small culture volumes will greatly facilitate the accomplishment of this objective.

In analysis of our APC data, we found that the search engine XProteo provided comparable or better results when compared with several other search engines. XProteo is especially well tuned for interpretation of MS and MS/MS spectra of singly charged ions generated in the MALDI process. The search engine is accessible through the Internet (www.xproteo.com).

The comprehensiveness of the analysis of the protein complexes by the protein digestion techniques depends on the ability to detect and identify every peptide from the complex protein mixtures. To achieve high coverage of the analysis, many of the current MS techniques use a powerful combination of liquid chromatography with tandem mass spectrometry to analyze the protein digests [10]–[12]. Collection and interpretation of multiple MS and tandem MS/MS spectra from a series of eluted peptides sometimes produce candidate peptides that can bear post-translational modifications. Such analysis, however, is frequently complicated by the difficulties in detecting the low abundant species co-eluted with more abundant ones. Our approach is not limited by the time constrains, and allows us to measures MS^n^ spectra of every observable or hypothesized species in the sample [29], maximizing the completeness of the performed analysis. Usually, we get more than 50 % percent of the analyzed ion peaks assigned to the identified proteins. The rest of the ion peaks represent the pool of species which is increasingly difficult to identify [12]. These species may originate from the original tryptic peptides as a result of fragmentation during the sample ionization process, or as the result of post-translational modifications. The later are exhaustively elucidated with high sensitivity, accuracy and speed using our modular mass spectrometric tool.

Our results confirm the principle of building a modular tool from multiple mass spectrometers. The flexibility of the modular approach allows us to use the strengths of each mass spectrometer for collecting additive information about a sample in a data-dependent manner. Although we used only MALDI mass spectrometers to demonstrate the feasibility of a modular tool, mass spectrometers operating with electrospray ion sources coupled to an HPLC system can also be combined in a modular tool [42]. This modular concept, based on the strengths of mass spectrometers operating with both MALDI and ESI, provides an alternative and complementary route for building powerful mass spectrometric tools for the biological research.

## Materials and Methods

### Mass spectrometers

Two mass spectrometers were combined in one tool according to the scheme shown in **Figure 1B**. One mass spectrometer is an orthogonal time-of flight prOTOF from Perkin Elmer. The second mass spectrometer is a vMALDI-Ion Trap from Thermo Electron Company. The combination was achieved by

1. Introducing an interchangeable MALDI target which can be accepted by the different MALDI instruments.
2. Writing several computer programs that create method files for measurements of MS/MS spectra in the vMALDI-IT mass spectrometer based on the measurements of m/z values of the precursors in the prOTOF mass spectrometer, in the most efficient data dependent way.

### MALDI magnetic target

Multiple MALDI targets are printed on a thin sheet of magnet-backed paper (Avery, Ink Jet Magnetic Sheets, 0.3 mm thickness) using a standard inkjet printer. The printed template was created using PC Draft (version 5.0.5, Microspot, Ltd). The printed target currently adopts a 384-well plate format, but this can be easily changed. We also print marks recognized by both mass spectrometers to align and calibrate the initial plate position (see supplementary **Figure S5** online). The target sheet is then laminated with a polyethylene film coated with a thin layer of Indium Tin Oxide (Sigma-Aldrich, product # 639281). This film is optically transparent and, at the same time, electrically conductive. Finally, the targets are cut apart and trimmed to measure 116 mm×77 mm. After washing each target several times using Kim-wipes soaked in acetonitrile and water, we magnetically attach one target to either of the plate adapters accepted by the different mass spectrometers (see the same figure). A commercial version of these targets will be available soon from Thermo Electron Company.

### Computer software

The MS spectra obtained in the prOTOF mass spectrometer were extracted from the data base of the instrument with the program “ProTOF extractor” (version 1.6 created by Markus Kalkum, Beckman Research Institute, City of Hope). The spectra then were analyzed with the “m/z” program (version 2002.10.01 by Ronald Beavis, Beavis Informatics Ltd., Canada), which helps to find and label the m/z values of the ion peaks in the MS spectrum. The m/z values of the precursor ions detected in each MS spectrum of a particular sample are stored in a text file. We use a computer program “AutoMSMS”, written in house using AutoIt Basic-like scripting language (www.autoitscript.com), to create MS/MS data acquisition methods for the vMALDI-ion trap from these text files, according to user-defined instrument parameters.

### Sample preparation

#### Yeast strains and growth conditions

All *S. cerevisiae* strains used in this work are from the yeast-TAP-fusion library, with MAT a, BY4741 background [43].

#### Protein Tagging

We developed a general strategy for re-tagging TAP tagged proteins [43] by replacing the TAP tag [44] with a 3xFLAG-hexahistidine (3xFLAG-6xH) tandem affinity tag (or any other tag, in general) using PCR-mediated homologous recombination. The procedure is described in the supplementary **Methods S1** section on line.

#### Immunopurification of the complexes

APC complexes were co-purified with Cdc16-3xFLAG-6xH protein, from one liter of BY4741 yeast cells grown to mid log-phase (∼2*10^7^ cells/ml). We estimate that we purify approximately several µg of the intact APC complexes from a 1 liter of the yeast cell culture (see supplementary **Figure S3**). The major steps of the purification protocol are depicted in **Fig. 2**. Briefly, the first step was performed by adding ∼5 mg of M-270 Epoxy Dynabeads (Invitrogen), with immobilized anti-Flag antibody (M2, Sigma), to 5–7 ml of a crude cell extract. The Dynabeads were coated with the antibody essentially as described in the manufacture's protocol, at ∼10 µg of antibody/5 mg of beads. After 30 min incubation, the beads were collected with a magnet and washed 3 times with 1 ml of IP buffer, 20 mM Hepes, 2 mM MgCl_2_, 250 mM NaCl, 0.1% tween, and protease inhibitor cocktail (Sigma-Aldrich). The enriched proteins were eluted with 300 µl of IP buffer containing a 3xFLAG peptide (Sigma-Aldrich), at a concentration of 200 µg/ml, for 30 min at 4°C whith constant rotation. The eluate was collected and diluted in 1 ml of IP buffer. The second purification step was performed with 20 µl of TALON Dynabeads (Invitrogen). The enriched proteins can be efficiently eluted with an SDS running buffer containing 250 mM imidazole and separated by SDS-PAGE. Alternatively, the purified protein complexes are left on the beads for on-bead digestion.

#### Digestion of the protein complexes on the beads

After the final wash of Talon beads with two times 1 ml of IP buffer and two times 1 ml of 50 mM ammonium bicarbonate buffer, the proteins were digested directly on the beads with 10 µl of trypsin solution (1 pmol/µl) in 10–50 mM ammonium bicarbonate buffer. After a brief initial digestion, the beads were separated from a supernatant with a magnet, and the solution was collected and left at 37 C for 5–6 hours to complete digestion.

### Sample preparation

1–3 µl of a mixture of either synthetic peptides or tryptic peptides were deposited on the interchangeable MALDI target and allowed to dry. 2 µl of a saturated solution of 4HCCA matrix was then added to the spot and again allowed to dry. The sample spots were then washed two times with 10% MeOH in 0.1% TFA by applying a 5–7 µl droplet on the top of the sample for 15–30 seconds and then quickly aspirating it.

### Nano-scale LC/MS/MS analysis of the APC complex

Approximately 100 ng of the digested APC preparation (∼1/10 portion of the total sample) were analyzed by nano-scale LC/MS/MS using a QTRAP mass spectrometer (Applied Biosystems, Foster City, USA) coupled to an LC Packings Ultimate/Famos/Switchos liquid chromatography system (Dionex). The sample was span down in an Eppendorf centrifuge at 13 krpm for 10 min to minimize the chance of introducing small magnetic micropaticles, which were not completely removed by the magnet and still were present in the solution. Peptides were transiently captured on a 0.3 mm by 5 mm C18 trap column before resolution over a 75 micron×150 mm C18 column. A two hour gradient of five to thirty-five percent acetonitrile was used with a constant concentration of 0.1% formic acid and a flow rate of 150 nl/min. Tandem mass spectra were acquired automatically in IDA mode using EMC survey scans. The resulting data were analysed with MASCOT (Matrix Science) on a local server.

### Additional Methods

Descriptions of the yeast strains and procedures for re-tagging of the TAP-tag with 3xFLAG-6xH tag are available in the supplementary **Methods S1.**

## Supporting Information

Methods S1 Descriptions of the yeast strains and procedures for re-tagging of the TAP-tag with 3xFLAG-6xH tag
(0.04 MB DOC)

Figure S1 Proteins identified after a time course incubation of trypsin solution with the cobalt-chelating beads containing the APC complexes.
(0.04 MB DOC)

Figure S2 Schematic diagram of the control experiment.
(0.09 MB DOC)

Figure S3 Summary of the proteins identified after SDS-PAGE separation of the affinity purified APC complexes.
(2.79 MB DOC)

Figure S4 MS/MS and MS/MS/MS (MS^3^) of the phosphopeptides detected in the APC proteins. Interpretation of the fragments in the MS/MS/MS spectra was performed with the assumption that the loss of the phosphate group (HPO_3_, MW. ∼80 Da) and water (∼18 Da) occurred from the same residue. Based on this assumption, we calculated the theoretical fragments of the peptides and compared these calculated fragments to the observed ones. Spectra are given in the increasing order of the m/z values of the detected phosphopeptides. See summary table in Figure 4C of the article.
(0.55 MB DOC)

Figure S5 Construction of the magnetic MALDI target.
(1.02 MB DOC)

Report S1 A report of the XProteo search engine (www.xproteo.com) containing information about components of the APC complexes identified with a modular mass spectrometric tool.
(1.44 MB DOC)

Report S2 A report of the XProteo search engine (www.xproteo.com) containing information about the proteins identified in the control immmunopurification experiment.
(0.47 MB DOC)

Report S3 A report of the Mascot search engine (www.matrixscience.com) containing information about components of the APC complexes identified with a QTRAP mass spectrometer (Sciex) coupled by online electrospray ionization to a nano-HPLC.
(5.32 MB DOC)

## References

[1] Yost R, Enke RK (1978). Selected ion fragmentation with a tandem quadrupole mass spectrometer.. J. Am. Chem. Soc..

[2] Yost RA, Boyd RK (1990). Tandem Mass spectrometry: quadrupole and hybrid instruments.. Methods Enzymol..

[3] Chernushevich IV, Loboda AV, Thomson BA (2001). An introduction to quadrupole-time-of-flight mass spectrometry.. J Mass Spectrom..

[4] Hager JW (2002). A new linear ion trap mass spectrometer. Rapid Commun.. Mass Spectrom..

[5] Vestal ML, Campbell JM (2005). Tandem time-of-flight mass spectrometry.. Methods Enzymol..

[6] Wu JT, Qian MG, Li MX, Liu L, Lubman DM (1996). Use of an ion trap storage/reflectron time-of-flight mass spectrometer as a rapid and sensitive detector for capillary electrophoresis in protein digest analysis.. Anal Chem..

[7] Martin RL, Brancia FL (2003). Analysis of high mass peptides using a novel matrix-assisted laser desorption/ionisation quadrupole ion trap time-of-flight mass spectrometer.. Rapid Commun Mass Spectrom..

[8] Syka JE, Marto JA, Bai DL, Senko MW, Horning S (2004). Novel linear quadrupole ion trap/FT mass spectrometer: performance characterization and use in the comparative analysis of histone H3 post-translational modifications.. J Proteome Res..

[9] Hu Q, Noll RJ, Li H, Makarov A, Hardman M (2005). The Orbitrap: a new mass spectrometer.. J Mass Spectrom..

[10] Martin SE, Shabanowitz J, Hunt DF, Marto JA (2000). Subfemtomole MS and MS/MS peptide sequence analysis using nano-HPLC micro-ESI fourier transform ion cyclotron resonance mass spectrometry.. Anal Chem..

[11] Olsen JV, de Godoy LM, Li G, Macek B, Mortensen P (2005). Parts per million mass accuracy on an Orbitrap mass spectrometer via lock mass injection into a C-trap.. Mol Cell Proteomics..

[12] Elias JE, Haas W, Faherty BK, Gygi SP (2005). Comparative evaluation of mass spectrometry platforms used in large-scale proteomics investigations.. Nature Methods.

[13] de Godoy LM, Olsen JV, de Souza GA, Li G, Mortensen P, Mann M (2006). Status of complete proteome analysis by mass spectrometry: SILAC labeled yeast as a model system.. Genome Biology.

[14] Ens W, Standing KG (2005). Hybrid quadrupole/time-of-flight mass spectrometers for analysis of biomolecules.. Methods Enzymol..

[15] Purves RW, Li L (1997). Development and characterization of an electrospray ionization ion trap/linear time-of-flight mass spectrometer.. J Am Soc Mass Spectrom.

[16] Makarov A, Denisov E, Kholomeev A, Balschun W, Lange O (2006). Performance evaluation of a hybrid linear ion trap/orbitrap mass spectrometer.. Anal Chem..

[17] Krutchinsky AN, Zhang W, Chait BT (2000). Rapidly switchable matrix-assisted laser desorption/ionization and electrospray quadrupole-time-of-flight mass spectrometry for protein identification.. J Am Soc Mass Spectrom..

[18] Krutchinsky AN, Kalkum M, Chait BT (2001). Automatic identification of proteins with a MALDI-quadrupole ion trap mass spectrometer.. Anal Chem..

[19] Su IH, Basavaraj A, Krutchinsky AN, Hobert O, Ullrich A (2003). Ezh2 controls B cell development through histone H3 methylation and Igh rearrangement.. Nat. Immunol..

[20] Ye JZ, Hockemeyer D, Krutchinsky AN, Loayza D, Hooper SM (2004). POT1-interacting protein PIP1: a telomere length regulator that recruits POT1 to the TIN2/TRF1 complex.. Genes Dev..

[21] Zhang X, Krutchinsky A, Fukuda A, Chen W, Yamamura S (2005). MED1/TRAP220 exists predominantly in a TRAP/ Mediator subpopulation enriched in RNA polymerase II and is required for ER-mediated transcription.. Mol Cell..

[22] Cristea IM, Williams R, Chait BT, Rout MP (2005). Fluorescent proteins as proteomic probes.. Mol Cell Proteomics.

[23] Loboda AV, Ackloo S, Chernushevich IV (2003). A high-performance matrix-assisted laser desorption/ionization orthogonal time-of-flight mass spectrometer with collisional cooling.. Rapid Commun Mass Spectrom..

[24] Garrett TJ, Yost RA (2006). Analysis of Intact Tissue by Intermediate-Pressure MALDI on a Linear Ion Trap Mass Spectrometer.. Anal. Chem..

[25] Peters JM (2006). The anaphase promoting complex/cyclosome: a machine designed to destroy.. Nat Rev Mol Cell Biol..

[26] Stafford G (2002). Ion trap mass spectrometry: a personal perspective.. J Am Soc Mass Spectrom..

[27] Schwartz JC, Senko MW, Syka JE (2002). A two dimentional quadrupole ion trap.. J Am Soc Mass Spectrom.

[28] Qin J, Chait BT (1997). Identification and Characterization of Posttranslational Modifications of Proteins by MALDI Ion Trap Mass Spectrometry.. Anal Chem..

[29] Chang EJ, Archambault V, McLachlin DT, Krutchinsky AN, Chait BT (2004). Analysis of protein phosphorylation by hypothesis-driven multiple-stage mass spectrometry.. Anal Chem..

[30] Schroeder M, Shabanowitz J, Schwartz JC, Hunt DF, Coon JJ (2004). A Neutral loss activation method for improved phosphopeptide sequence analysis by quadrupole ion trap mass spectrometry.. Anal Chem..

[31] Fenyo D, Qin J, Chait BT (1998). Protein identification using mass spectrometric information.. Electrophoresis..

[32] Knop M, Siegers K, Pereira G, Zachariae W, Winsor B (1999). Epitope tagging of yeast genes using a PCR-based strategy: more tags and improved practical routines.. Yeast.

[33] Rigaut G, Shevchenko A, Rutz B, Wilm M, Mann M, Seraphin B (1999). A generic protein purification method for protein complex characterization and proteome exploration.. Nat. Biotechnol..

[34] Robeva AS, Woodart R, Lutchin DR, Taylor HE, Linden J (1996). Double tagging recombinant A1 and A2A-adenosine receptors with hexahistidine and the FLAG epitope.. Biochem. Pharmacology.

[35] Sadygov RG, Cociorva D, Yates JR (2004). Large-scale database searching using tandem mass spectra: looking up the answer in the back of the book.. Nat Methods..

[36] Odegrip R, Coomber D, Eldridge B, Hederer R, Kuhlman PA (2004). CIS display: In vitro selection of peptides from libraries of protein-DNA complexes.. Proc Natl Acad Sci U S A..

[37] Tackett AJ, DeGrasse JA, Sekedat MD, Oeffinger M, Rout MP, Chait BT (2005). I-DIRT, a general method for distinguishing between specific and nonspecific protein interactions.. J Proteome Res..

[38] Phizicky EM, Fields S (1995). Protein-protein interactions: methods for detection and analysis.. Microbiol Rev..

[39] Annan RS, Carr SA (1996). Phosphopeptide analysis by matrix assisted laser desorption time-of-flight mass spectrometry.. Anal. Chem..

[40] Rudner AD, Murray AW (2000). Phosphorylation by Cdc28 activates the Cdc20-dependent activity of the anaphase-promoting complex.. J. Cell Biol..

[41] Kraft C, Herzog F, Gieffers C, Mechtler K, Hagting A (2003). Mitotic regulation of the human anaphase-promoting complex by phosphorylation.. EMBO J..

[42] Bassmann C, Lubeck M, Gebhardt C, Ledertheil T (2006). Mass spectrometric mixture analysis, United States Patent Application 20060289737.

[43] Ghaemmaghami S, Huh WK, Bower K, Howson RW, Belle A (2003). Global analysis of protein expression in yeast.. Nature..

[44] Puig O, Caspary F, Rigaut G, Rutz B, Bouveret E (2001). The tandem affinity purification (TAP) method: a general procedure of protein complex purification.. Methods..

