# Supplementary material for: Modular Mass Spectrometric Tool for Analysis of Composition and Phosphorylation of Protein Complexes
Source: PLoS One. 2007 Apr 4;2(4):e358. doi: 10.1371/journal.pone.0000358 (PMC1832223; doi:10.1371/journal.pone.0000358)
Supplement: Methods S1 — Descriptions of the yeast strains and procedures for re-tagging of the TAP-tag with 3xFLAG-6xH tag (0.04 MB DOC) [file pone.0000358.s001.doc]

**Supplementary Methods S1**

**Yeast strains and growth conditions**

All *Saccharomyces cerevisiae* strains used in this work are from the yeast-TAP-fusion library , with MAT a, BY4741 background [43].

**Replacement of a TAP tag**

The tandem affinity purification (TAP) tag in each yeast strain expressing the protein of interest was replaced with a 3xFLAG-Hexahistitine (DYKDHDGDYKDHDIDYKDDDDKHHHHH) double tag. The major steps of the re-tagging procedure are depicted in the figure.

**I. Modification of pUG72 vector.**

Vector pUG72 (Güldener U, Heinisch J, Köhler GJ, Voss D, and Hegemann JH. (2002) A second set of loxP marker cassettes for Cre-mediated multiple gene knockouts in budding yeast. Nucleic Acids Research, 30**,** e23) was obtained from Euroscarf. The pUG72 vector was modified by inserting a 3xFLAG-Hx6-(STOP) construct, GACTACAAGGACCATGACGGTGATTACAAGGATCATGACATCGACTACAAGGATGACGATGACAAGCATCATCACCATCACCATGGTTGACTGCA, in the proximity of a *Kluyveromyces lactis* URA3 selection marker.

The DNA sequence of the modified pUG72-3xFLAG-6xH vector is

TGACACATGCAGCTCCCGGAGACGGTCACAGCTTGTCTGTAAGCGGATGCCGGGAGCAGACAAGCCCGTCAGGGCGCGTCAGCGGGTGTTGGCGGGTGTCGGGGCTGGCTTAACTATGCGGCATCAGAGCAGATTGTACTGAGAGTGCACCATATGGACATATTGTCGTTAGAACGCGGCTACAATTAATACATAACCTTATGTATCATACACATACGATTTAGGTGACACTATAGAACGCGGCCGCCAGGACTACAAGGACCATGACGGTGATTACAAGGATCATGACATCGACTACAAGGATGACGATGACAAGCATCATCACCATCACCATGGTTGACTGCAGGTCGACAACCCTTAATATAACTTCGTATAATGTATGCTATACGAAGTTATTAGGTCTAGAGATCCCAATACAACAGATCACGTGATCTTTTGTAAGATGAAGTTGAAGTGAGTGTTGCACCGTGCCAATGCAGGTGGCTATTAGATTAAATATGTGATTTGTTCTATTAAGTTTCCTGTATAATTAATGGGGAGCGCTGATTCTCTTTTGGTACGCTTCCCATCCAGCATTTCTGTATCTTTCACCTTCAACCTTAGGATCTCTACCCTTGGCGAAAAGTCCTCTGCCAACAATGATGATATCTGATCCACCACTTACAACTTCGTCGACGGTTCTGTACTGCTGACCCAATGCATCGCCTTTGTCGTCTAAACCTACACCTGGGGTCATGATTAGCCAATCAAACCCTTCTTCTCTTCCTCCCATATCGTTCTGAGCAATGAACCCAATAACGAAATCTTTATCACTCTTTGCAATATCAACGGTACCCTTAGTATATTCACCGTGTGCTAGAGAACCCTTGGAAGACAATTCAGCAAGCATCAATAATCCCCTTGGTTCTTTGGTGACCTCTTGCGCACCTTGTTTCAAGCCAGCAACAATACCAGCACCAGTAACCCCGTGGGCGTTGGTGATATCAGACCATTCTGCGATACGGTAAACGCCCGATGTATATTGTAATTTGACTGTGTTACCGATATCGGCGAATTTTCTGTCCTCAAATATCAAGAACTTGTATTTCTCTGCCAATGCTTTCAATGGAACGACAGTACCCTCATAACTGAAATCATCCAAGATATCAACGTGTGTTTTCAAAAGGCAAATGTATGGACCCAACGTTTCAACAAGTTTCAATAGCTCATCAGTCGAACGAACGTCAAGAGAAGCACACAAATTGGTCTTCTTTTCATCCATTAAACGTAAAAGTTTCGATGCAACCGGACTTGCATGAGTCTCAGCTCTACTGGTATATGATTTTGTGGACATGGTGCAACTAATTGACGGGAGTGTATTGACGCTGGCGTACTGGCTTTCACAAAATGGCCCAATCACAACCACATCTTAGATAGTTGAAATGACTTTAGATAACATCAATTGAGATGAGCTTAATCATGTCAAAGCTAAAAGTGTCACCATGAACGACAATTCTTAAGCAAATCACGTGATATAGATCCACGAATAACCACCATTTGATGCTCGAGGCAAGTAATGTGTGTAAAAAAATGCGTTACCACCATCCAATGCAGACCGATCTTCTACCCAGAATCACATATATTTATGTACCGAGTACCTTTTTTCTATCTTCCAATTGCTTCTCCCATATGATTGTCTCCGTAAGCTCGAAATTTCTAAGTTGGATTTTAATCTTCACGCAGGATGACAGTTCGATGAGCTTCTGAGGAGTGTTTAGAACATAATCAGTTTATCCATGGTCTATCTCTTCTTGTCGCTTTTTCTCCTCGATAGAACCTAAATAAAACGAGCTCTCGAGAACCCTTAATATAACTTCGTATAATGTATGCTATACGAAGTTATTAGGTGATATCAGATCCACTAGTGGCCTATGCGGCCGCGGATCTGCCGGTCTCCCTATAGTGAGTCGTATTAATTTCGATAAGCCAGGTTAACCTGCATTAATGAATCGGCCAACGCGCGGGGAGAGGCGGTTTGCGTATTGGGCGCTCTTCCGCTTCCTCGCTCACTGACTCGCTGCGCTCGGTCGTTCGGCTGCGGCGAGCGGTATCAGCTCACTCAAAGGCGGTAATACGGTTATCCACAGAATCAGGGGATAACGCAGGAAAGAACATGTGAGCAAAAGGCCAGCAAAAGGCCAGGAACCGTAAAAAGGCCGCGTTGCTGGCGTTTTTCCATAGGCTCCGCCCCCCTGACGAGCATCACAAAAATCGACGCTCAAGTCAGAGGTGGCGAAACCCGACAGGACTATAAAGATACCAGGCGTTTCCCCCTGGAAGCTCCCTCGTGCGCTCTCCTGTTCCGACCCTGCCGCTTACCGGATACCTGTCCGCCTTTCTCCCTTCGGGAAGCGTGGCGCTTTCTCAATGCTCACGCTGTAGGTATCTCAGTTCGGTGTAGGTCGTTCGCTCCAAGCTGGGCTGTGTGCACGAACCCCCCGTTCAGCCCGACCGCTGCGCCTTATCCGGTAACTATCGTCTTGAGTCCAACCCGGTAAGACACGACTTATCGCCACTGGCAGCAGCCACTGGTAACAGGATTAGCAGAGCGAGGTATGTAGGCGGTGCTACAGAGTTCTTGAAGTGGTGGCCTAACTACGGCTACACTAGAAGGACAGTATTTGGTATCTGCGCTCTGCTGAAGCCAGTTACCTTCGGAAAAAGAGTTGGTAGCTCTTGATCCGGCAAACAAACCACCGCTGGTAGCGGTGGTTTTTTTGTTTGCAAGCAGCAGATTACGCGCAGAAAAAAAGGATCTCAAGAAGATCCTTTGATCTTTTCTACGGGGTCTGACGCTCAGTGGAACGAAAACTCACGTTAAGGGATTTTGGTCATGAGATTATCAAAAAGGATCTTCACCTAGATCCTTTTAAATTAAAAATGAAGTTTTAAATCAATCTAAAGTATATATGAGTAAACTTGGTCTGACAGTTACCAATGCTTAATCAGTGAGGCACCTATCTCAGCGATCTGTCTATTTCGTTCATCCATAGTTGCCTGACTCCCCGTCGTGTAGATAACTACGATACGGGAGGGCTTACCATCTGGCCCCAGTGCTGCAATGATACCGCGAGACCCACGCTCACCGGCTCCAGATTTATCAGCAATAAACCAGCCAGCCGGAAGGGCCGAGCGCAGAAGTGGTCCTGCAACTTTATCCGCCTCCATCCAGTCTATTAATTGTTGCCGGGAAGCTAGAGTAAGTAGTTCGCCAGTTAATAGTTTGCGCAACGTTGTTGCCATTGCTACAGGCATCGTGGTGTCACGCTCGTCGTTTGGTATGGCTTCATTCAGCTCCGGTTCCCAACGATCAAGGCGAGTTACATGATCCCCCATGTTGTGCAAAAAAGCGGTTAGCTCCTTCGGTCCTCCGATCGTTGTCAGAAGTAAGTTGGCCGCAGTGTTATCACTCATGGTTATGGCAGCACTGCATAATTCTCTTACTGTCATGCCATCCGTAAGATGCTTTTCTGTGACTGGTGAGTACTCAACCAAGTCATTCTGAGAATAGTGTATGCGGCGACCGAGTTGCTCTTGCCCGGCGTCAATACGGGATAATACCGCGCCACATAGCAGAACTTTAAAAGTGCTCATCATTGGAAAACGTTCTTCGGGGCGAAAACTCTCAAGGATCTTACCGCTGTTGAGATCCAGTTCGATGTAACCCACTCGTGCACCCAACTGATCTTCAGCATCTTTTACTTTCACCAGCGTTTCTGGGTGAGCAAAAACAGGAAGGCAAAATGCCGCAAAAAAGGGAATAAGGGCGACACGGAAATGTTGAATACTCATACTCTTCCTTTTTCAATATTATTGAAGCATTTATCAGGGTTATTGTCTCATGAGCGGATACATATTTGAATGTATTTAGAAAAATAAACAAATAGGGGTTCCGCGCACATTTCCCCGAAAAGTGCCACCTGACGTCTAAGAAACCATTATTATCATGACATTAACCTATAAAAATAGGCGTATCACGAGGCCCTTTCGTCTCGCGCGTTTCGGTGATGACGGTGAAAACCTC

**II. PCR of the replacement cassette**

The cassette for replacement of a TAP-tag-His3MX insert was produced by PCR using the modified pUG72-3xFLAG-6xH vector as a template. Phusion™ Hot Start High-Fidelity DNA Polymerase and HF buffer (F-540S, New England Biolabs, Ipswich, MA) were used to amplify the replacement cassette. The PCR primer pair, 5’-ATT CCA ACT ACT GCT AGC GAG AAT TTG TAT TTT CAG GGA GAA TTC GGC CTT TAC GAT TTA GGT GAC ACT ATA GAA-3’(sense) and 5’-GAT GAA TTC GAG CTC GTT TAA ACT GGA TGG CGG CGT TAG TAT CGA ATC GAC TAA TAC GAC TCA CTA TAG GGA GAC-3’(anti-sense), were synthesized by Integrated DNA Technologies (Coralville, IA). The PCR was performed as follows:. Incubation at 98℃ for 5 minutes, followed by 35 cycles of 30 seconds at 98℃, 30 seconds at 60℃ and 5 minutes at 72℃, then incubation at 72℃ for 10 minutes and holding at 4℃.

The first 50 base pairs of the TAP replacing cassette has a DNA sequence identical to the DNA sequence of a calmodulin binding peptide (CBP) followed by the TEV protease cleavage site [44]. The last 50 base pairs of the TAP replacing cassette have a DNA sequence identical to the part of the DNA sequence of a His3MX selection marker cassette (Longtine M.S. http://opbs.okstate.edu/~longtine/homepage.html). The resulting sequence of the 3xFlag-6xHis-URA3 cassette for replacement of a TAP tag is

ATTCCAACTACTGCTAGCGAGAATTTGTATTTTCAGGGAGAATTCGGCCTT TACGATTTAGGTGACACTATAGAACGCGGCCGCCAGGACTACAAGGACCATGACGGTGATTACAAGGATCATGACATCGACTACAAGGATGACGATGACAAGCATCATCACCATCACCATGGTTGACTGCAGGTCGACAACCCTTAATATAACTTCGTATAATGTATGCTATACGAAGTTATTAGGTCTAGAGATCCCAATACAACAGATCACGTGATCTTTTTGTAAGATGAAGTTGAAGTAGTGTTGCACCGTGCCAATGCAGGTGGCTATTAGATTAAATATGTGATTTGTTCTATTAAGTTTCCTGTATAATTAATGGGGAGCGCTGATTCTCTTTTGGTACGCTTCCCATCCAGCATTTCTGTATCTTTCACCTTCAACCTTAGGATCTCTACCCTTGGCGAAAAGTCCTCTGCCAACAATGATGATATCTGATCCACCACTTACAACTTCGTCGACGGTTCTGTACTGCTGACCCAATGCATCGCCTTTGTCGTCTAAACCTACACCTGGGGTCATGATTAGCCAATCAAACCCTTCTTCTCTTCCTCCCATATCGTTCTGAGCAATGAACCCAATAACGAAATCTTTATCACTCTTTGCAATATCAACGGTACCCTTAGTATATTCACCGTGTGCTAGAGAACCCTTGGAAGACAATTCAGCAAGCATCAATAATCCCCTTGGTTCTTTGGTGACCTCTTGCGCACCTTGTTTCAAGCCAGCAACAATACCAGCACCAGTAACCCCGTGGGCGTTGGTGATATCAGACCATTCTGCGATACGGTAAACGCCCGATGTATATTGTAATTTGACTGTGTTACCGATATCGGCGAATTTTCTGTCCTCAAATATCAAGAACTTGTATTTCTCTGCCAATGCTTTCAATGGAACGACAGTACCCTCATAACTGAAATCATCCAAGATATCAACGTGTGTTTTCAAAAGGCAAATGTATGGACCCAACGTTTCAACAAGTTTCAATAGCTCATCAGTCGAACGAACGTCAAGAGAAGCACACAAATTGGTCTTCTTTTCATCCATTAAACGTAAAAGTTTCGATGCAACCGGACTTGCATGAGTCTCAGCTCTACTGGTATATGATTTTGTGGACATGGTGCAACTAATTGACGGGAGTGTATTGACGCTGGCGTACTGGCTTTCACAAAATGGCCCAATCACAACCACATCTTAGATAGTTGAAATGACTTTAGATAACATCAATTGAGATGAGCTTAATCATGTCAAAGCTAAAAGTGTCACCATGAACGACAATTCTTAAGCAAATCACGTGATATAGATCCACGAATAACCACCATTTGATGCTCGAGGCAAGTAATGTGTGTAAAAAAATGCGTTACCACCATCCAATGCAGACCGATCTTCTACCCAGAATCACATATATTTATGTACCGAGTACCTTTTTTCTATCTTCCAATTGCTTCTCCCATATGATTGTCTCCGTAAGCTCGAAATTTCTAAGTTGGATTTTAATCTTCACGCAGGATGACAGTTCGATGAGCTTCTGAGGAGTGTTTAGAACATAATCAGTTTATCCATGGTCTATCTCTTCTTGTCGCTTTTTCTCCTCGATAGAACCTAAATAAAACGAGCTCTCGAGAACCCTTAATATAACTTCGTATAATGTATGCTATACGAAGTTATTAGGTGATATCAGATCCACTAGTGGCCTATGCGGCCGCGGATCTGCCGGTCTCCCTATAGTGAGTCGTATTAGTCGATTCGATACTAACGCCGCCATCCAGTTTAAACGAGCTCGAATTCATC

**III. Homologous recombination**

The PCR product mixture was directly used for homologous recombination using a transformation protocol essentially as described by Gietz RD and Woods RA. (2002) Transformation of yeast by lithium acetate/single-stranded carrier DNA/polyethylene glycol method. Methods Enzymol., 350, 87-96.

**IV. Selection**

The re-tagged yeast cells were selected on plates lacking uracil in the growth medium. After the re-tagging procedure, the resulting C-terminal tag on the proteins is

…..GRRIPGLINPWKRRWKKNFIAVSAANRFKKISSSGALDYDIPTTASENLYFQGEFGLYDLGDTIERGRQDYKDHDGDYKDHDIDYKDDDDKHHHHHHG(Stop) corresponding to CBP-TEV-3xFlag-6xHis tag.
