# Supplementary material for: Modular Mass Spectrometric Tool for Analysis of Composition and Phosphorylation of Protein Complexes
Source: PLoS One. 2007 Apr 4;2(4):e358. doi: 10.1371/journal.pone.0000358 (PMC1832223; doi:10.1371/journal.pone.0000358)
Supplement: Figure S1 — Proteins identified after a time course incubation of trypsin solution with the cobalt-chelating beads containing the APC complexes. (0.04 MB DOC) [file pone.0000358.s002.doc]

**Figure S1**

**(A)** Proteins identified after a time course incubation of trypsin solution with the cobalt-chelating beads containing the APC complexes. The experiment was performed in the following way:

**Step 1**. 10 μl of trypsin solution were added to the washed beads and incubated at 37 C for 0.5 min. The supernatant was removed from the beads by magnetic separation and, incubated at 37C for 5 hours. Proteins eluted during short incubation with trypsin were digested for 5 hours and identified according to the method described in the paper. Eight proteins, all from the APC complexes, were identified in this sample.

**Step 2**. We added another 10 μl of the trypsin solution to the same beads and incubated for 5 min at 37 C. The supernatant was removed from the beads, and the eluted proteins were digested for 5 hours at 37C. Thirteen proteins from APC complexes were identified in this sample. The detailed protein identification results are presented in the XProteo supplementary **Report S1**.

**Step 3**. We added another 10 μl of the trypsin solution to the same beads and incubated for 50 min at 37 C. The supernatant was removed from the beads, and the eluted proteins were digested for 5 hours. Nine APC proteins were identified in this sample.

**Step 4**. Finally, we added 10 μl of trypsin solution for the last time and incubated it with the beads for 5 hours. This final step produced identification of three proteins, *Cdc27, Cdc23* and *Bud27*. The last protein is probably an impurity.

**(B)** Proteins identified in a separate experiment with the immunopurified APC complexes. The initial conditions for this experiment were similar to the initial conditions of the experiment described in **A**, except a 10 μl of solution trypsin was added to the cobalt beads and incubated at 37C for 5 hours. 17 proteins were identified in the supernatant after digestion. Among all the proteins, 13 proteins are from the APC complexes. The other 4 proteins, *Uba4, Ssa1, Cdc19* and *Pho84* are presumably the impurities, which are frequently identified in the control experiments.
