# Supplementary material for: Modular Mass Spectrometric Tool for Analysis of Composition and Phosphorylation of Protein Complexes
Source: PLoS One. 2007 Apr 4;2(4):e358. doi: 10.1371/journal.pone.0000358 (PMC1832223; doi:10.1371/journal.pone.0000358)
Supplement: Figure S2 — Schematic diagram of the control experiment. (0.09 MB DOC) [file pone.0000358.s003.doc]

**Identification of proteins in the control IP experiment**

**Figure S2.** The control experiment performed in the way identical to the principal experiment, with an equal amount of yeast cells of a background strain (BY4147, MAT a) lacking any tagged protein. Analysis of prOTOF MALDI-MS spectrum resulted in detection of 236 ion peaks with the signal-to-noise above 1.2. 236 MS/MS spectra of the detected precursors were obtained in the vMALDI-IT mass spectrometer. The data were converted into the DTA format and supplied to the XProteo search engine. This procedure resulted in identification of several impurities. The identified proteins, the number of peptides and the protein coverage are indicated in the table. For additional information, see supplementary **Report S2**.
