# Supplementary material for: Modular Mass Spectrometric Tool for Analysis of Composition and Phosphorylation of Protein Complexes
Source: PLoS One. 2007 Apr 4;2(4):e358. doi: 10.1371/journal.pone.0000358 (PMC1832223; doi:10.1371/journal.pone.0000358)
Supplement: Figure S3 — Summary of the proteins identified after SDS-PAGE separation of the affinity purified APC complexes. (2.79 MB DOC) [file pone.0000358.s004.doc]

**Figure S3.** Summary of the proteins identified after SDS-PAGE separation of the affinity purified APC complexes in the microGel (Life-Gels, Life Therapeutics, Australia) stained with a colloidal coomassie stain (GelCode, Pierce). Samples were obtained after tandem affinity purification of APC complexes from ~(2-4)x10^10^ yeast cells containing

**(+)** a Cdc16 protein tagged at the C-terminus with 3xFLAG–hexahistidine tags and;

**(-)** no proteins with the affinity tags (left gel lane, control experiment).

We estimate that we purify approximately several μg of the intact APC complexes from a 1 liter of the yeast cell culture. Each gel lane was chopped onto 14 pieces and the proteins were identified according to a standard in-gel digestion procedure (see, for example [21]).
