# Supplementary material for: Modular Mass Spectrometric Tool for Analysis of Composition and Phosphorylation of Protein Complexes
Source: PLoS One. 2007 Apr 4;2(4):e358. doi: 10.1371/journal.pone.0000358 (PMC1832223; doi:10.1371/journal.pone.0000358)
Supplement: Figure S5 — Construction of the magnetic MALDI target. (1.02 MB DOC) [file pone.0000358.s006.doc]

**Figure S5.** Magnetic target is attached to **(A)** the magnetic steal plate 117x77mm, 0.61 mm thickness, whish is inserted in a standard plate adapter of the prOTOF mass spectrometer **(B)** magnetic steal plate mounted on the top of a modified vMALDI-IT plate adapter. Arrows indicate the position of marks recognized by the different mass spectrometers, and used for calibrating the MALDI target position.
