# Supplementary material for: Modular Mass Spectrometric Tool for Analysis of Composition and Phosphorylation of Protein Complexes
Source: PLoS One. 2007 Apr 4;2(4):e358. doi: 10.1371/journal.pone.0000358 (PMC1832223; doi:10.1371/journal.pone.0000358)
Supplement: Report S1 — A report of the XProteo search engine (www.xproteo.com) containing information about components of the APC complexes identified with a modular mass spectrometric tool. (1.44 MB DOC) [file pone.0000358.s007.doc]

**Report S1**.

A report of the XProteo search engine ([www.xproteo.com](http://www.xproteo.com/)) containing information about components of the APC complexes identified with a modular mass spectrometric tool.

The APC complexes were purified as described in the paper. The proteins co-purified with Cdc16-3xFLAG-6xH were digested on the beads. The tryptic mixture was analyzed directly without any fractionation step in the prOTOF mass spectrometer. First, we measured accurate m/z values of 319 ion peaks detected in the MALDI MS spectrum, with the signal-to-noise above 1.2. Then we measured MS/MS spectra of all 319 peaks in the vMALDI ion trap mass spectrometer. We used accurate values of m/z values of the precursor ions together with the structural information obtained from MS/MS spectra to perform search for *Saccharomyces cerevisiae* proteins (NCBI nonredundant data base version 06/06/07) with the Xproteo search engine utilizing

the following search parameters:

Protein mass: **0.0~300.0kDa**  Mixture search: **Auto**

Protein pI: **1.0~14.0**  Instrument: **MALDI_I_TRAP**

Enzyme: **Trypsin** Mass type: **Monoisotopic**

Max. missed cleavage: **Auto** Charge state: **1+**

Modifications: Precursor errors: **0.030Da**

**(C)None; (P)None** Fragment errors: **0.300Da**

XProteo first ranks candidate proteins by probability scores calculated from
an improved version of the Bayesian algorithm used in ProFound (Zhang W, and Chait B.T BT. (2000) ProFound: an expert system for protein identification using mass spectrometric peptide mapping information. Anal. Chem. **72**, 2482-2489). XProteo then calculates discriminability (d') for each candidate protein, defined as the normalized distance between the score distributions of the identified protein and randomly matched proteins in units of standard deviation of the score distributions.

**The following pages of the Report show:**

The summary of the results on the top identified proteins…………………. Pages 2-4

Results of identification of Apc1 protein……………………………………… Pages 5-6

Results of identification of Cdc16 protein ……………………………........... Pages 7-8

Result of identification of Cdc27 protein……………………………….......... Pages 9-10

Result of identification of Cdc23 protein……………………………………… Pages 11-12

Result of identification of Apc9 protein………………………… ……………. Pages 13-14

Result of identification of Apc2 protein……………………………………….. Pages 15-16

Result of identification of Mnd2 protein………………………………………. Pages 17

Result of identification of Cdc26 protein……………………………………… Page 18

Result of identification of Apc5 protein……………………………………….. Pages 19-20

Result of identification of Doc1 protein………………………………………. Page 21

Result of identification of Apc4 protein………………………………………. Pages 22-23

Result of identification of Swm1 protein……………………………………… Page 24

Result of identification of Apc11 protein……………………………….. ……. Pages 25

Interpretation of the two MS/MS spectra

of two tryptic peptides from Apc11 protein…………………………………… Pages26-27
