# Supplementary material for: Modular Mass Spectrometric Tool for Analysis of Composition and Phosphorylation of Protein Complexes
Source: PLoS One. 2007 Apr 4;2(4):e358. doi: 10.1371/journal.pone.0000358 (PMC1832223; doi:10.1371/journal.pone.0000358)
Supplement: Report S2 — A report of the XProteo search engine (www.xproteo.com) containing information about the proteins identified in the control immmunopurification experiment. (0.47 MB DOC) [file pone.0000358.s008.doc]

**Report S2**.

A report of the XProteo search engine ([www.xproteo.com](http://www.xproteo.com/)) containing information about the proteins identified in the control immmunopurification experiment.

The control experiment was performed according to the scheme depicted in **Supplementary Figure 1**, with the yeast strain (BY4147, MAT a) lacking any tagged protein. Proteins, which adhere to the beads in some non-specific fashion, were digested with trypsin and the tryptic mixture was analyzed directly without any fractionation step in the prOTOF mass spectrometer. 236 ion peaks were detected in the MALDI MS spectrum, with the signal-to-noise above 1.2. Then we measured MS/MS spectra of all 236 peaks in the vMALDI-ion trap mass spectrometer. We used accurate values of m/z values of the precursor ions together with the structural information obtained from MS/MS spectra to perform search for *Saccharomyces cerevisiae* proteins (NCBI non-redundant data base version 10/16/06) with the Xproteo search engine utilizing the following search parameters:

Protein mass: **0.0~300.0kDa**  Mixture search: **Auto**

Protein pI: **1.0~14.0**  Instrument: **MALDI_I_TRAP**

Enzyme: **Trypsin** Mass type: **Monoisotopic**

Max. missed cleavage: **Auto** Charge state: **1+**

Modifications: Precursor errors: **0.030Da**

**(C)None; (P)None** Fragment errors: **0.300Da**

XProteo first ranks candidate proteins by probability scores calculated from
an improved version of the Bayesian algorithm used in ProFound (Zhang, W. and Chait, B.T B.T. ProFound: an expert system for protein identification using mass spectrometric peptide mapping information. *Anal. Chem.* **72**, 2482-2489 (2000)). XProteo then calculates discriminability (d') for each candidate protein, defined as the normalized distance between the score distributions of the identified protein and randomly matched proteins in units of standard deviation of the score distributions.

**The following pages of the Report show:**

The summary of the results on the top identified proteins………….. Pages 2-3

Results of identification of Fks1 protein………………………………. Pages 4-5

Results of identification of Tef1 protein ………………………………. Pages 6-7

Result of identification of Pho84 protein………………………………..Pages 8

Result of identification of Adh1protein…………………………………. Pages 9
