# Supplementary material for: Modular Mass Spectrometric Tool for Analysis of Composition and Phosphorylation of Protein Complexes
Source: PLoS One. 2007 Apr 4;2(4):e358. doi: 10.1371/journal.pone.0000358 (PMC1832223; doi:10.1371/journal.pone.0000358)
Supplement: Report S3 — A report of the Mascot search engine (www.matrixscience.com) containing information about components of the APC complexes identified with a QTRAP mass spectrometer (Sciex) coupled by online electrospray ionization to a nano-HPLC. (5.32 MB DOC) [file pone.0000358.s009.doc]

**Report S3**.

A report of the Mascot search engine ([www.matrixscience.com](http://www.xproteo.com/)) containing information about components of the APC complexes identified with a QTRAP mass spectrometer (Sciex) coupled by online electrospray ionization to a nano-HPLC.

The APC complexes were purified as described in the paper. The proteins co-purified with Cdc16-3xFLAG-6xH were digested on the beads. Approximately 1/10^th^ of the final sample was analyzed by the nano-HPLC QTRAP mass spectrometer described in the **Materials and Methods** section.

**Search Parameters**

Type of search :**MS/MS Ion Search**

Enzyme :**Trypsin**

Mass values :**Monoisotopic**

Protein Mass :**Unrestricted**

Peptide Mass Tolerance : **+/- 2 Da**

Fragment Mass Tolerance : **+/- 0.8 Da**

Max Missed Cleavages : **2**

Instrument type : **ESI-QTRAP plus internals**

Number of queries : **573**
