# Supplementary material for: Modular Mass Spectrometric Tool for Analysis of Composition and Phosphorylation of Protein Complexes
Source: PLoS One. 2007 Apr 4;2(4):e358. doi: 10.1371/journal.pone.0000358 (PMC1832223; doi:10.1371/journal.pone.0000358)
Supplement: Supplementary file 9 [file pone.0000358.s005.doc]

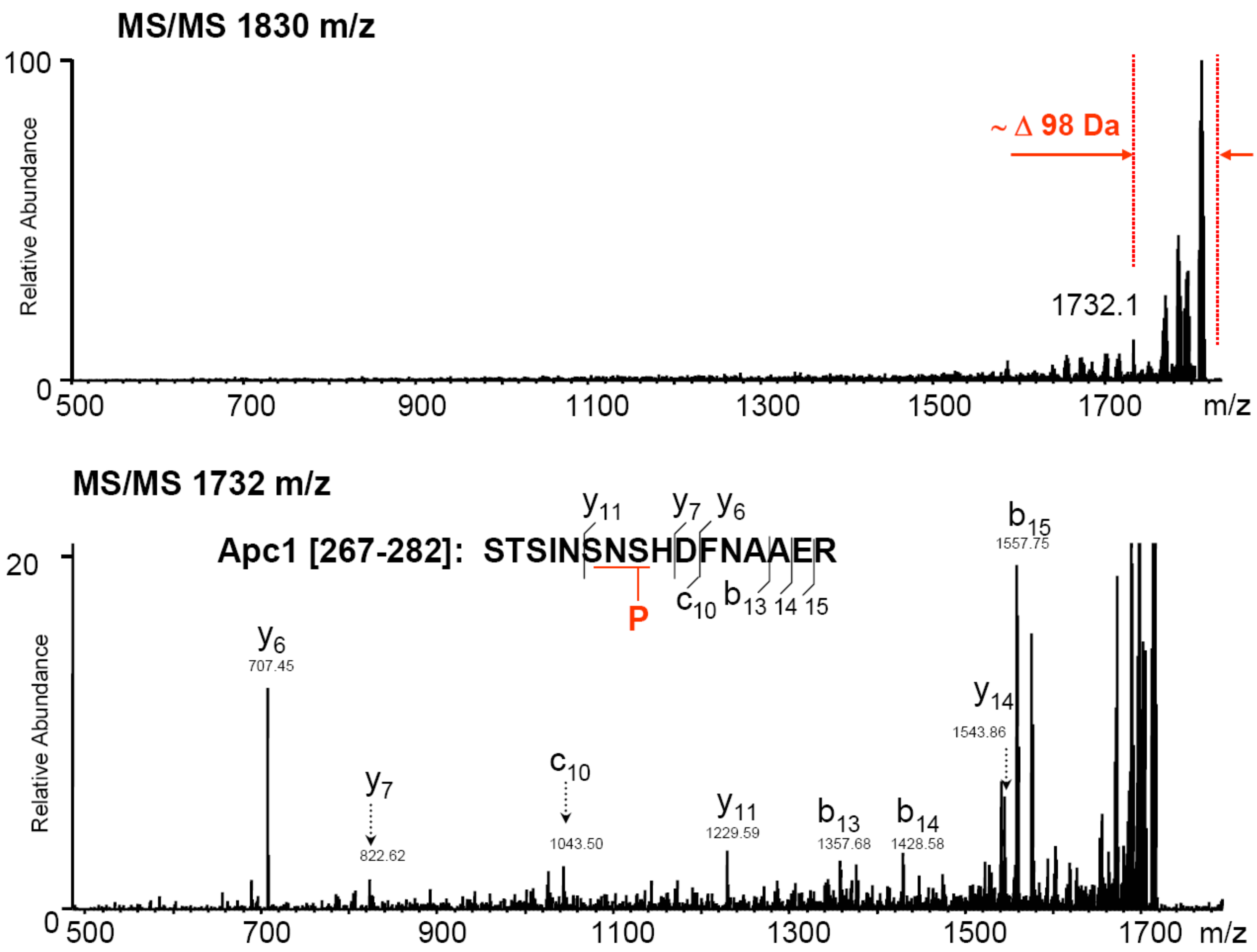
MS/MS 1830 m/z
~ Δ 98 Da
1732.1
Relative Abundance
m/z
MS/MS 1732 m/z
Apc1 [267-282]: STSINSNSHDFNAAER
P
y11 y7 y6
c10 b13 14 15
y6 707.45
y7 822.62
c10 1043.50
y11 1229.59
b13 1357.68
b14 1428.58
y14 1543.86
b15 1557.75
Relative Abundance
m/z

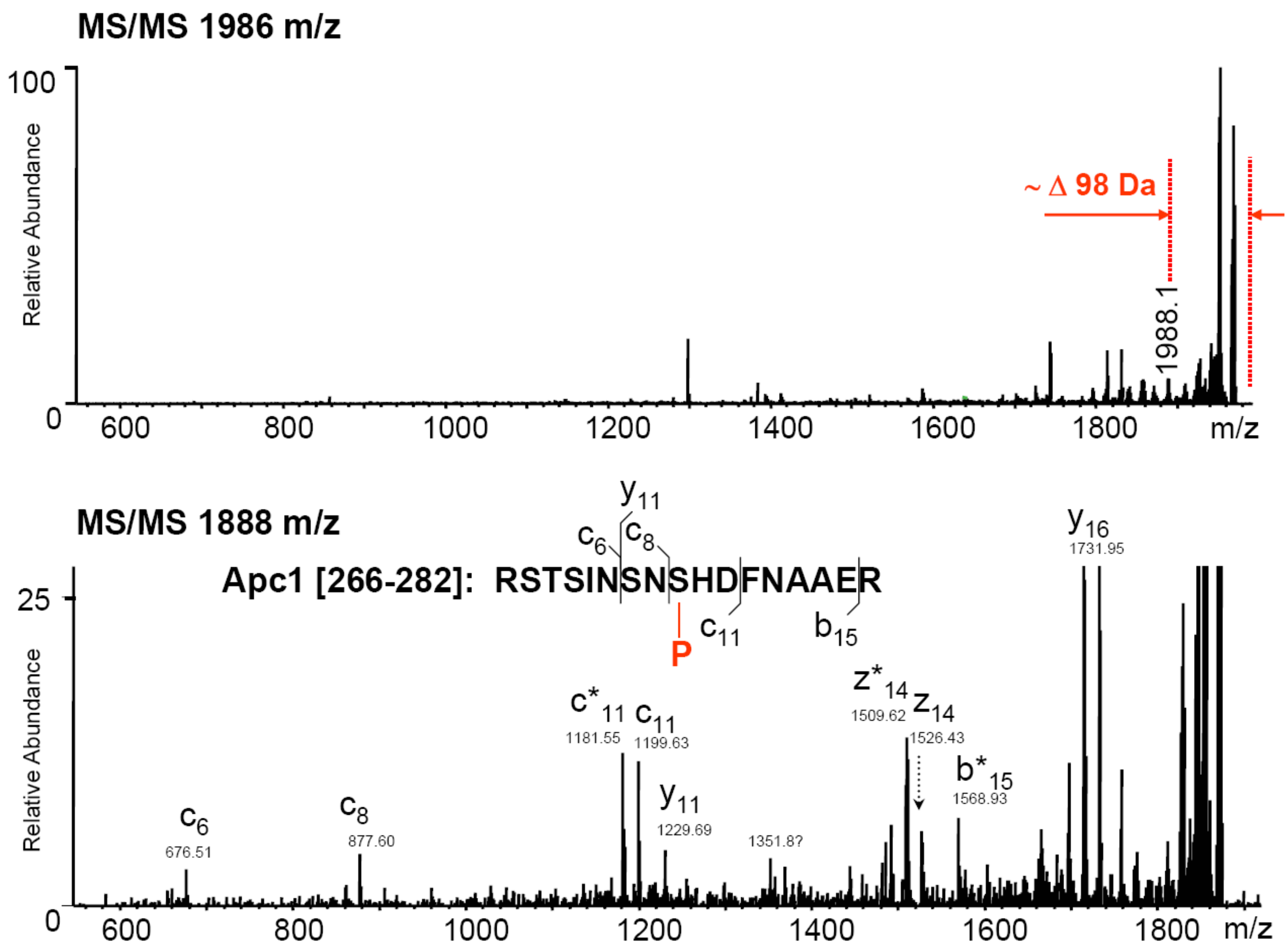

MS/MS 1986 m/z
100
Relative Abundance
0
~ Δ 98 Da
1988.1
600
800
1000
1200
1400
1600
1800
m/z
MS/MS 1888 m/z
Apc1 [266-282]: RSTSINSNSHDFNAAER
P
y11
c6
c8
c11
b15
y16
1731.95
25
c*11
1181.55
c11
1199.63
y11
1229.69
1351.87
z*14
1509.62
z14
1526.43
b*15
1568.93
c6
676.51
c8
877.60
0

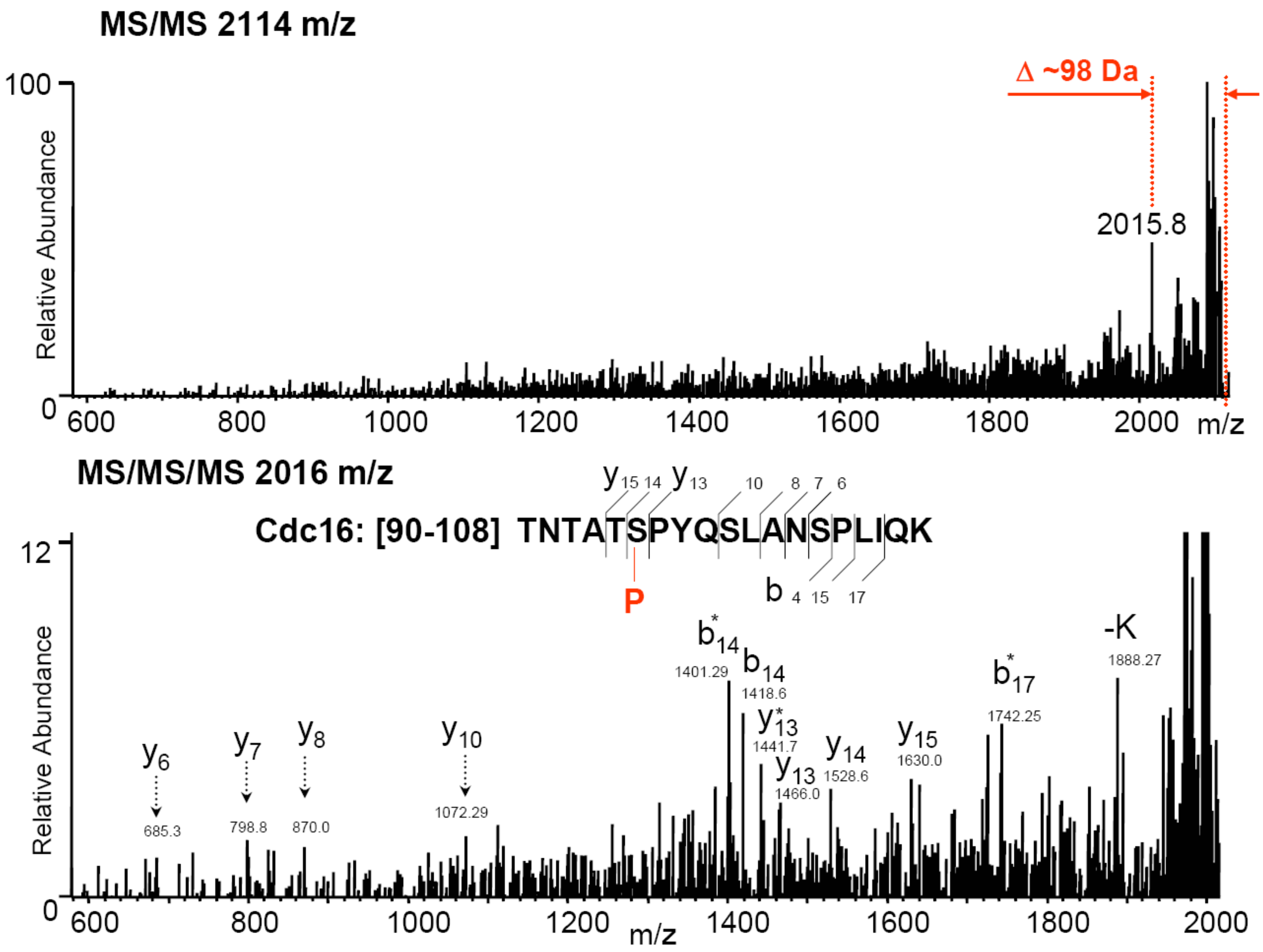

MS/MS 2114 m/z
Δ ~98 Da
2015.8
Relative Abundance
m/z
MS/MS/MS 2016 m/z
Cdc16: [90-108] TNTATSPYQSLANSPLIQK
P
-K
1888.27
b*14 1401.29
b14 1418.6
y*13 1441.7
y13 1466.0
y14 1528.6
y15 1630.0
b*17 1742.25
y6 685.3
y7 798.8
y8 870.0
y10 1072.29

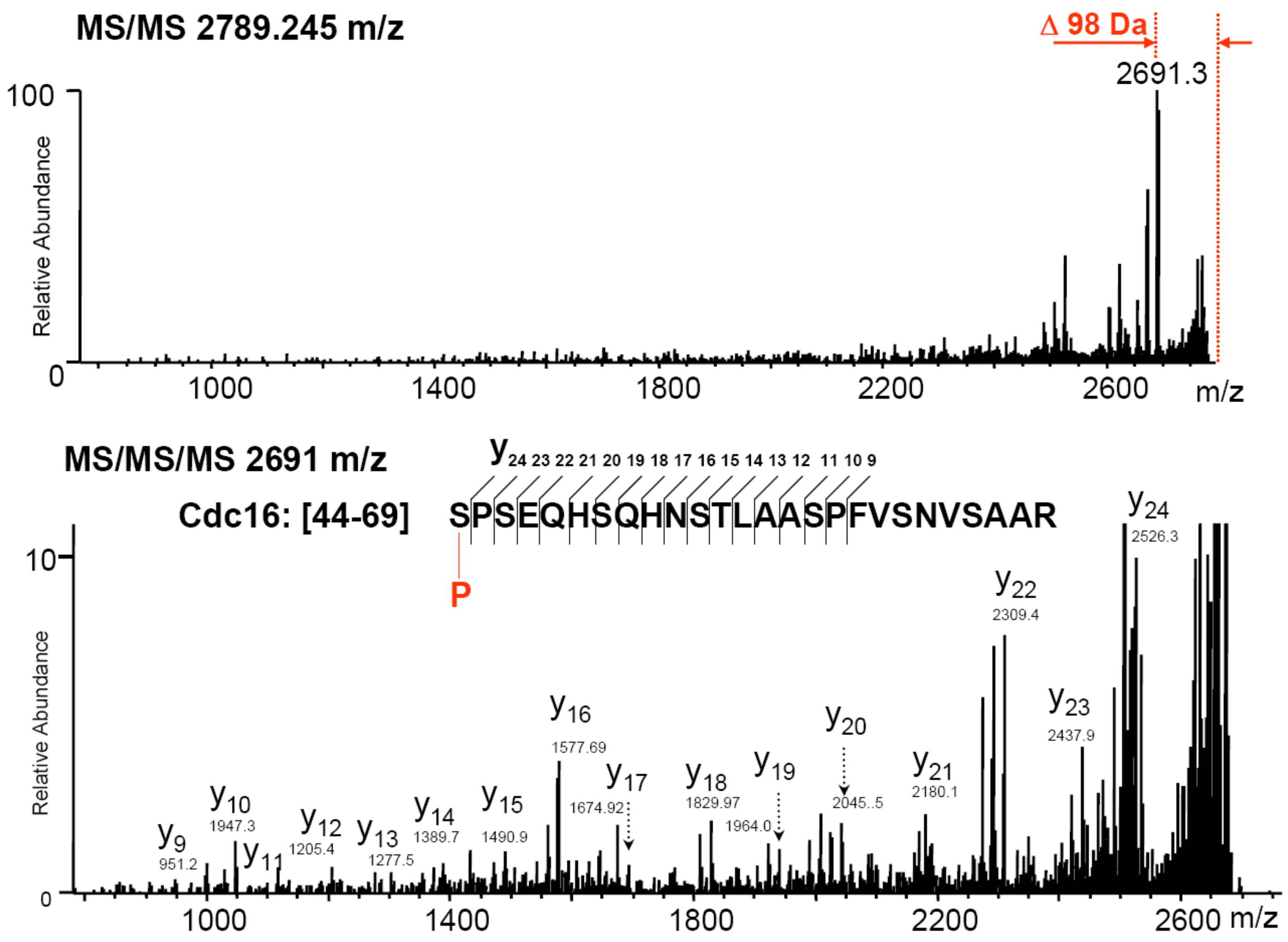

MS/MS 2789.245 m/z
Δ 98 Da
2691.3
Relative Abundance
100
0
1000
1400
1800
2200
2600
m/z
MS/MS/MS 2691 m/z
Cdc16: [44-69]
SPSEQHSQHNSTLAASPFVSNVSAAR
P
y24 23 22 21 20 19 18 17 16 15 14 13 12 11 10 9
y24
2526.3
y22
2309.4
y23
2437.9
y21
2180.1
y20
2045..5
y19
1964.0
y18
1829.97
y17
1674.92
y16
1577.69
y15
1490.9
y14
1389.7
y13
1277.5
y12
1205.4
y11
y10
1947.3
y9
951.2
10

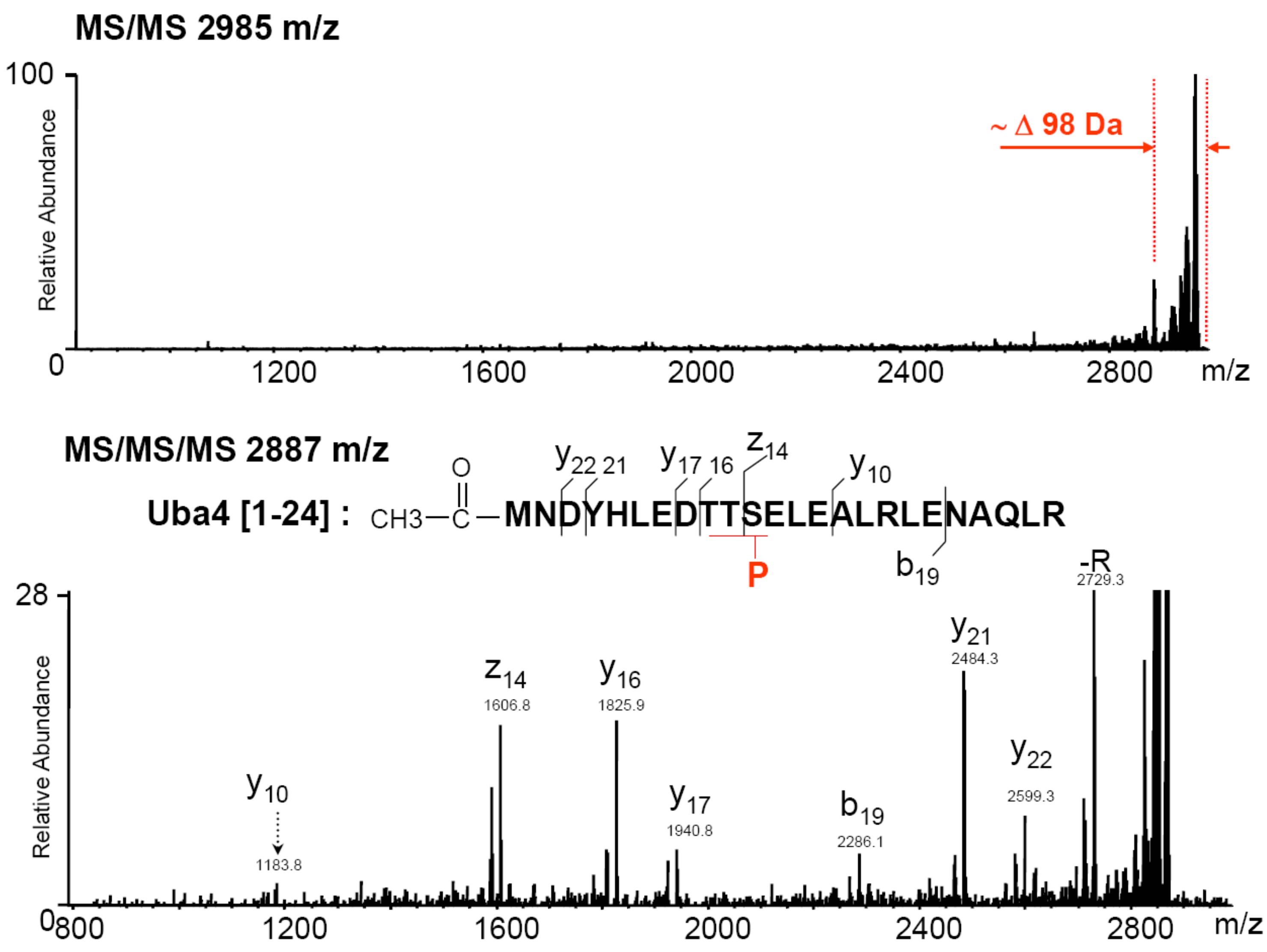
MS/MS 2985 m/z
~ Δ 98 Da
Relative Abundance
100
0
1200
1600
2000
2400
2800
m/z
MS/MS/MS 2887 m/z
Uba4 [1-24] : CH3—C(=O)—MNDYHLEDTTSELEALRLENAQLR
y22 21
y17 16
z14
y10
b19
P
28
0
-R
2729.3
y21
2484.3
z14
1606.8
y16
1825.9
y22
2599.3
y10
1183.8
y17
1940.8
b19
2286.1
800
1200
1600
2000
2400
2800
m/z

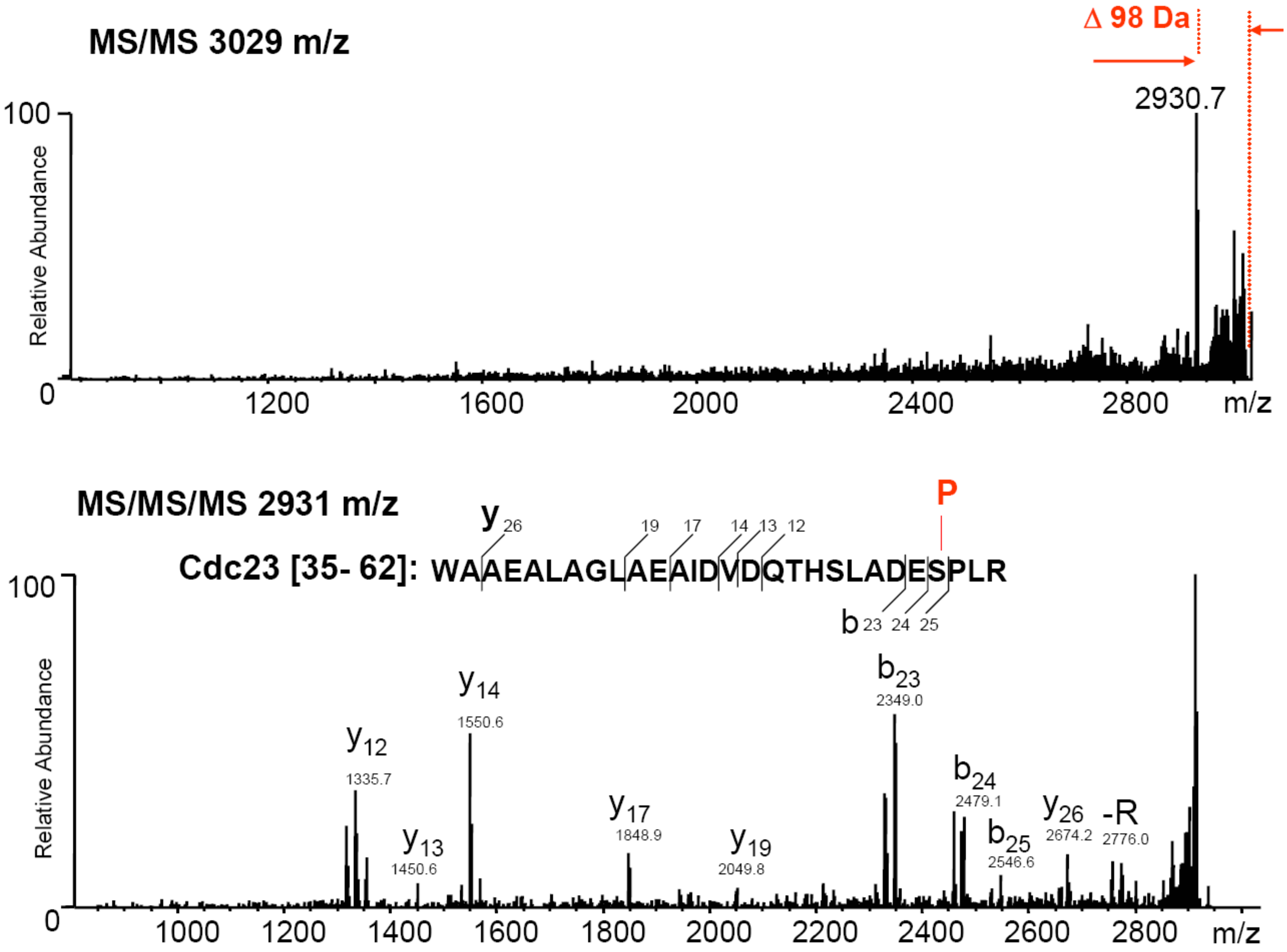

MS/MS 3029 m/z
Δ 98 Da
2930.7
Relative Abundance
100
0
1200
1600
2000
2400
2800
m/z
MS/MS/MS 2931 m/z
P
y 26 19 17 14 13 12
Cdc23 [35- 62]: WAAEALAGLAEAIDVDQTHSLADESPLR
b 23 24 25
Relative Abundance
100
0
y12 1335.7
y13 1450.6
y14 1550.6
y17 1848.9
y19 2049.8
b23 2349.0
b24 2479.1
b25 2546.6
y26 2674.2
-R 2776.0
1000
1200
1400
1600
1800
2000
2200
2400
2600
2800
m/z

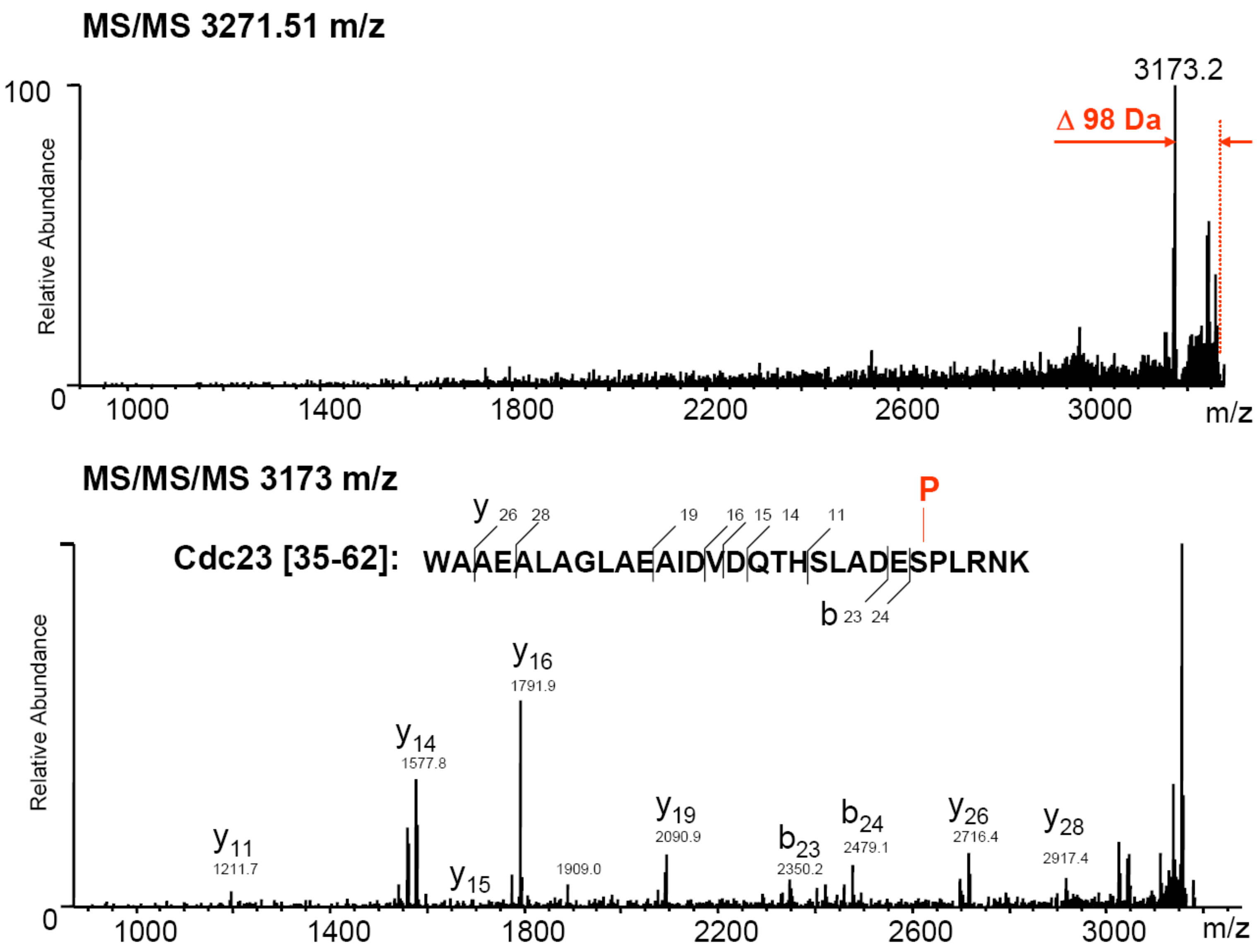
MS/MS 3271.51 m/z
3173.2
Δ 98 Da
Relative Abundance
100
0
1000
1400
1800
2200
2600
3000
m/z
MS/MS/MS 3173 m/z
Cdc23 [35-62]: WAAEALAGLAEAIDVDQTHSLADESPLRNK
P
y 26 28 19 16 15 14 11
b 23 24
y16 1791.9
y14 1577.8
y11 1211.7
y15
1909.0
y19 2090.9
b23 2350.2
b24 2479.1
y26 2716.4
y28 2917.4

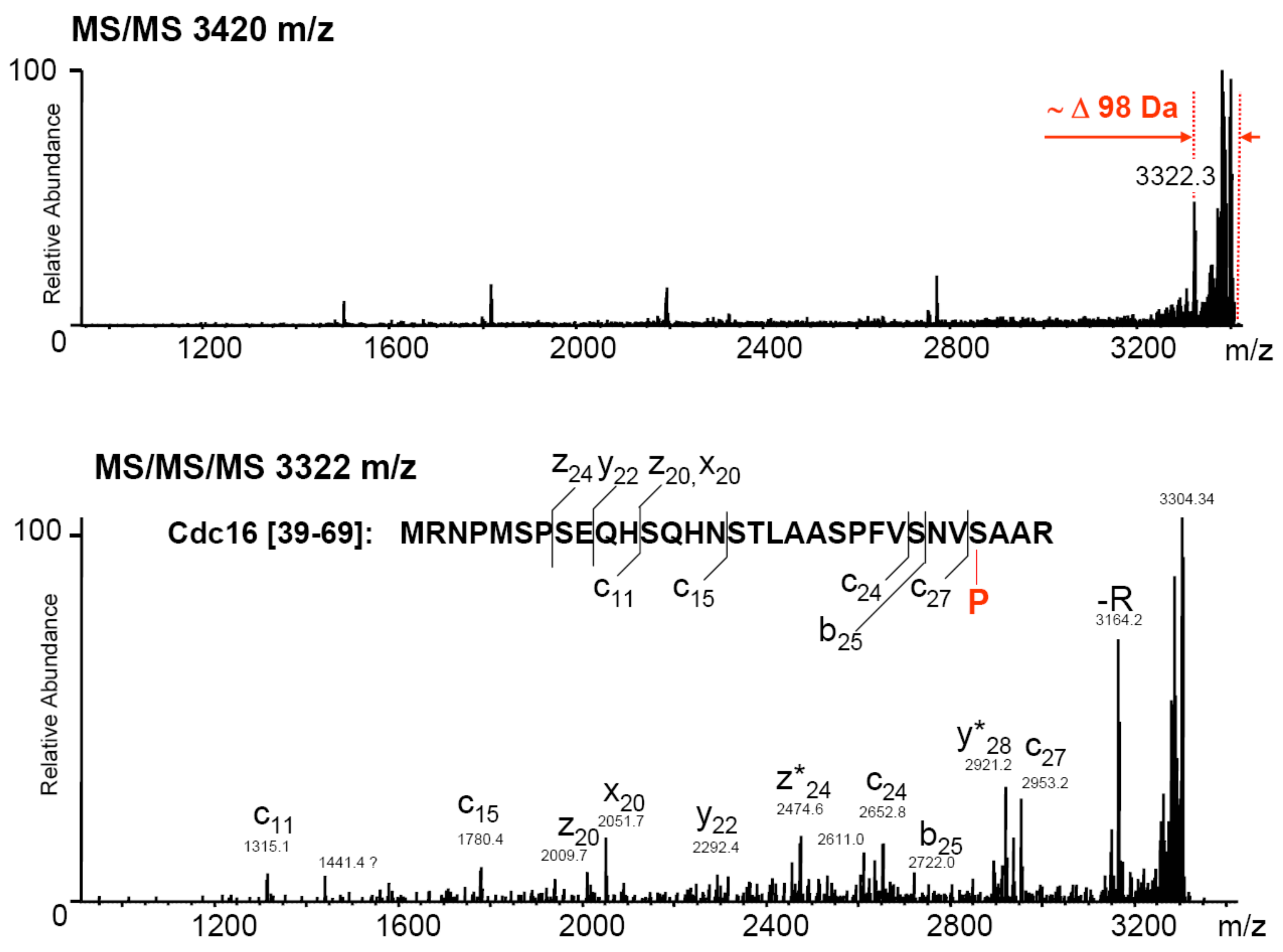
MS/MS 3420 m/z
100
Relative Abundance
0
~ Δ 98 Da
3322.3
1200
1600
2000
2400
2800
3200
m/z
MS/MS/MS 3322 m/z
Cdc16 [39-69]: MRNPMSPSEQHSQHNSTLAASPFVSNVSAAR
z24 y22 z20, x20
c11 c15 c24 c27 b25
P
3304.34
-R
3164.2
y*28 2921.2
c27 2953.2
z*24 2474.6
c24 2652.8
2611.0
b25 2722.0
y22 2292.4
x20 2051.7
z20 2009.7
c15 1780.4
c11 1315.1
1441.4 ?
100
Relative Abundance
0
1200
1600
2000
2400
2800
3200
m/z

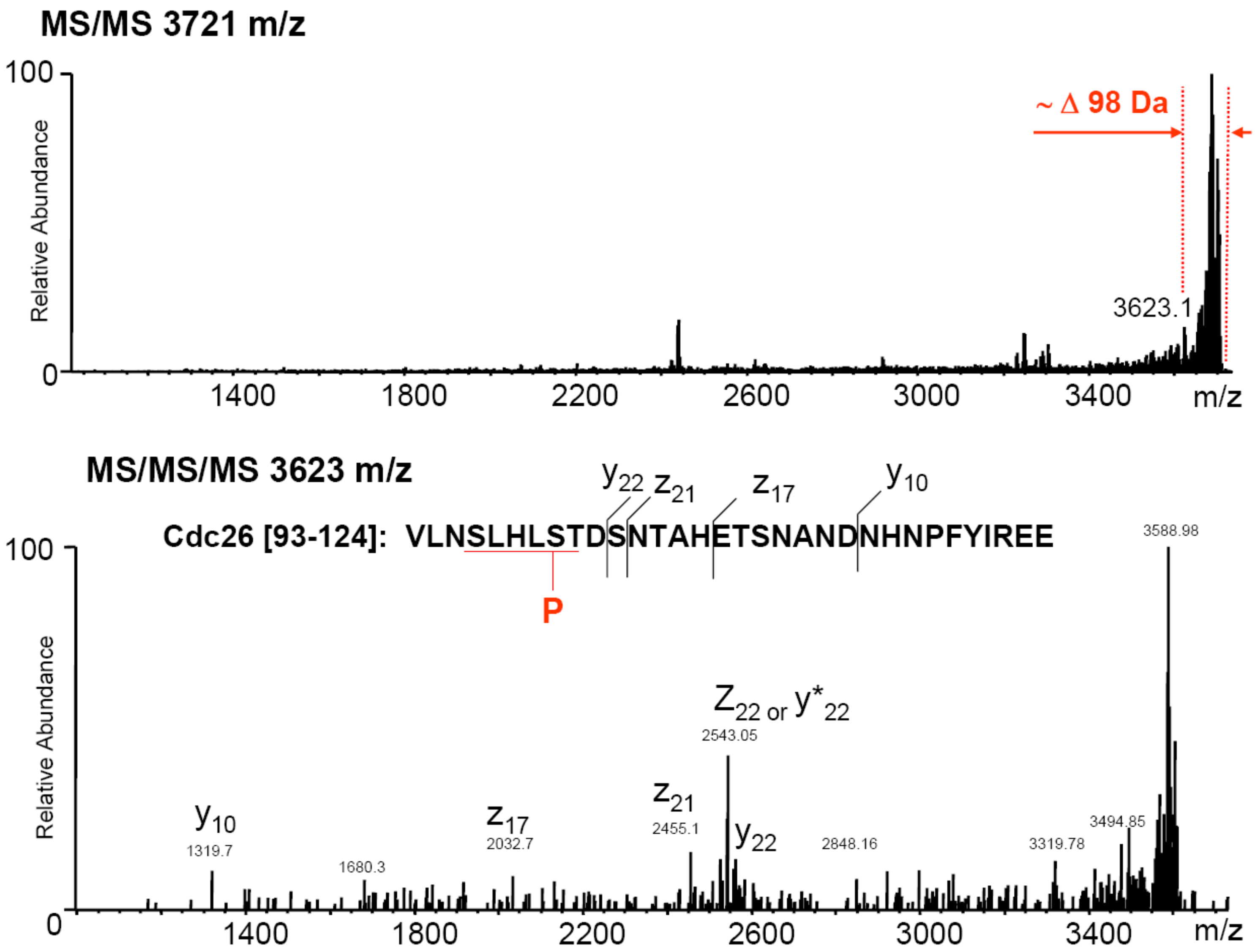

MS/MS 3721 m/z
100
0
Relative Abundance
~ Δ 98 Da
3623.1
1400
1800
2200
2600
3000
3400
m/z
MS/MS/MS 3623 m/z
Cdc26 [93-124]: VLNSLHLSTDSNTAHETSNANDNHNPFYIREE
P
y22
z21
z17
y10
3588.98
Z22 or y*22
2543.05
z21
2455.1
y22
2848.16
3319.78
3494.85
z17
2032.7
1680.3
y10
1319.7

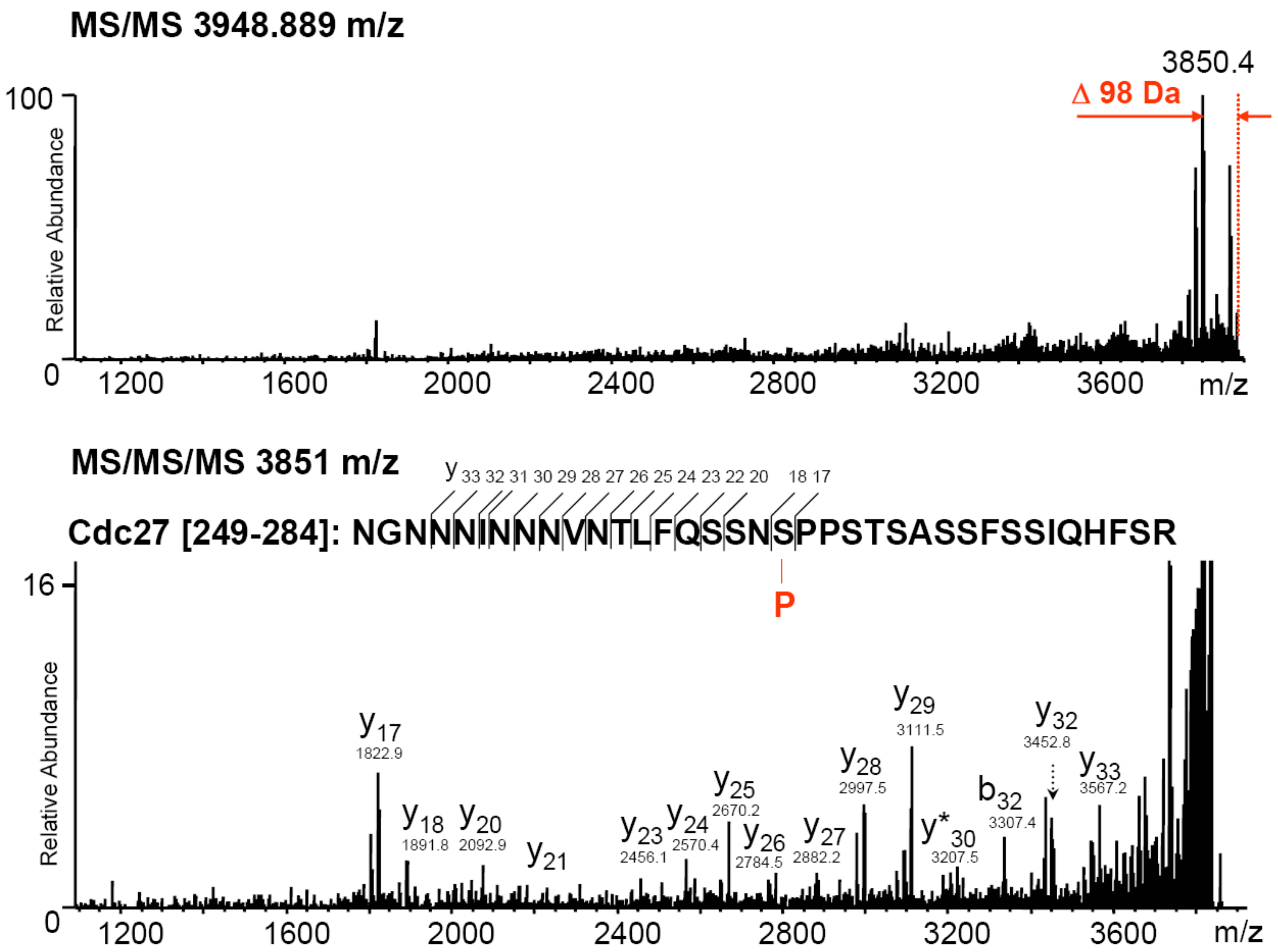

MS/MS 3948.889 m/z
3850.4
Δ 98 Da
Relative Abundance
100
0
1200
1600
2000
2400
2800
3200
3600
m/z
MS/MS/MS 3851 m/z
y 33 32 31 30 29 28 27 26 25 24 23 22 20 18 17
Cdc27 [249-284]: NGNNNINNNVNTLFQSSNSPPSTSASSFSSIQHFSR
P
16
y17 1822.9
y18 1891.8
y20 2092.9
y21
y23 2456.1
y24 2570.4
y25 2670.2
y26 2784.5
y27 2882.2
y28 2997.5
y29 3111.5
y*30 3207.5
b32 3307.4
y32 3452.8
y33 3567.2
